# Comprehensive lipid and lipid-related gene investigations of host immune responses to characterize metabolism-centric biomarkers for pulmonary tuberculosis

**DOI:** 10.1038/s41598-022-17521-4

**Published:** 2022-08-04

**Authors:** Nguyen Phuoc Long, Nguyen Ky Anh, Nguyen Thi Hai Yen, Nguyen Ky Phat, Seongoh Park, Vo Thuy Anh Thu, Yong-Soon Cho, Jae-Gook Shin, Jee Youn Oh, Dong Hyun Kim

**Affiliations:** 1 Department of Pharmacology and PharmacoGenomics Research Center, Inje University College of Medicine, Busan, Republic of Korea; 2 Center for Personalized Precision Medicine of Tuberculosis, Inje University College of Medicine, Busan, Republic of Korea; 3 School of Mathematics, Statistics and Data Science, Sungshin Women’s University, Seoul, Republic of Korea; 4 Department of Clinical Pharmacology, Inje University Busan Paik Hospital, Busan, Republic of Korea; 5 Division of Pulmonary, Allergy and Critical Care Medicine, Department of Internal Medicine, Korea University Guro Hospital, Seoul, Republic of Korea

**Keywords:** Diagnostic markers, Tuberculosis, Lipidomics

## Abstract

Despite remarkable success in the prevention and treatment of tuberculosis (TB), it remains one of the most devastating infectious diseases worldwide. Management of TB requires an efficient and timely diagnostic strategy. In this study, we comprehensively characterized the plasma lipidome of TB patients, then selected candidate lipid and lipid-related gene biomarkers using a data-driven, knowledge-based framework. Among 93 lipids that were identified as potential biomarker candidates, ether-linked phosphatidylcholine (PC O–) and phosphatidylcholine (PC) were generally upregulated, while free fatty acids and triglycerides with longer fatty acyl chains were downregulated in the TB group. Lipid-related gene enrichment analysis revealed significantly altered metabolic pathways (e.g., ether lipid, linolenic acid, and cholesterol) and immune response signaling pathways. Based on these potential biomarkers, TB patients could be differentiated from controls in the internal validation (random forest model, area under the curve [AUC] 0.936, 95% confidence interval [CI] 0.865–0.992). PC(O-40:4), PC(O-42:5), PC(36:0), and PC(34:4) were robust biomarkers able to distinguish TB patients from individuals with latent infection and healthy controls, as shown in the external validation. Small changes in expression were identified for 162 significant lipid-related genes in the comparison of TB patients vs. controls; in the random forest model, their utilities were demonstrated by AUCs that ranged from 0.829 to 0.956 in three cohorts. In conclusion, this study introduced a potential framework that can be used to identify and validate metabolism-centric biomarkers.

## Introduction

Tuberculosis (TB), a communicable disease caused by *Mycobacterium tuberculosis *(*Mtb*), remains a global health crisis. The World Health Organization (WHO) estimated that TB was responsible for 1.5 million deaths and approximately 10 million new patients in 2020^1^. The persistently high incidence and prevalence of TB in part reflect inadequate diagnostic approaches; it has been estimated that only 60% of new cases are detected, especially in countries with a high disease burden and low treatment coverage^1^. A sensitive and easy-to-implement test would provide an important initial improvement in diagnostic accuracy. However, the current standard in the diagnosis of TB is smear microscopy or culture tests, both of which have a low sensitivity, are laborious, and require a specialist laboratory^2^. Molecular tests, such as the Xpert MTB/RIF assay, have been introduced; however, their use is not economically feasible in primary care settings^3^. Moreover, TB tests often rely on sputum and thus have sub-optimal sensitivity, especially for patients with early active TB—such patients cannot consistently provide sputum^4^. To improve TB healthcare quality, the WHO has urged the development of a rapid and sensitive biomarker-based non-sputum test that can be implemented at the clinical site and utilizes accessible samples, such as blood, urine, or breath condensate^4^.

Omics-based discovery studies in TB patients—which involved comprehensive profiling of the host transcriptome, metabolome, and proteome—identified several biomarkers for the diagnosis of TB^5–11^. Moreover, implementing network analysis with multi-omics data could potentially verify and choosing the best among those biomarkers^12^. Transcriptomics is the most matured technology that identifies promising transcript biosignatures for TB diagnosis, treatment monitoring, and outcome prediction. Among the proposed signatures, Sweeney3, a host-response three-gene signature, has met the WHO’s target product profiles for a triage test^13^, whereas lipidomics research applications in TB management remain limited. Consequently, there is a need for further research, particularly into the altered lipidome of TB patients; lipids and lipid-related genes also have potential for use as diagnostic and prognostic biomarkers. Moreover, large-scale lipid profiling using plasma can provide insights into the disease because host lipids constitute a significant nutrition source for *Mtb* growth and reproduction^14^.

Mutual metabolic alterations constitute important aspects of host–pathogen interactions; together with regulatory factors, such alterations are responsible for drug tolerance but can be exploited to design effective host-tailored therapies^15,16^. *Mtb* lipid metabolism in host macrophages has a vital role in TB pathogenesis^16,17^. Lipid droplet (LD) formation, an important event in *Mtb* lipid metabolism, is a multifaceted process related to *Mtb* intracellular growth and drug tolerance; it also acts as a host defense mechanism to combat the pathogen^17–20^. Accordingly, studies that examine biomarkers related to lipid metabolism and immunology are expected to be fruitful.

There have been several investigations of the biological fluid lipidome in TB patients, with the goal of identifying biomarkers for TB diagnosis^21–25^. Chen et al. described changes in lipid levels during TB treatment, and the unbiased lipidomics approach of Shivakoti et al. revealed an association between the host lipidome and treatment failure^26,27^. These pioneering studies indicate significant differences in lipid profiles of patients with active TB and their counterparts; thus, they highlight potential applications of lipid and lipid-gene biomarkers in diverse clinical scenarios.

In the current study, a robust workflow was developed that facilitates the identification and validation of multi-omics metabolism-centric lipid and lipid-gene biomarkers for the diagnosis of active pulmonary TB.

## Material and methods

### Institutional review board statement for the clinical cohort

The Institutional Review Board of Korea University Guro Hospital reviewed and approved the study (No. 2017GR0012). All procedures were carried out following the Declaration of Helsinki. Written informed consent was obtained from all participants that allowed the blood and clinical data analysis to be used.

### Sample characteristics

As mentioned elsewhere, plasma and clinical data were obtained from the Biobank of Korea University Guro Hospital^28^. Patients with malignant diseases, diabetes mellitus, hyperlipidemia, human immunodeficiency virus infection, and chronic liver or renal diseases were excluded. Thus, 35 patients with confirmed pulmonary TB and 37 controls were included in this study. The demographic information of included populations was described in Supplementary Table S1. There were no statistically significant differences between the two groups in terms of age (Wilcoxon rank sum test) or sex (Fisher’s exact test).

### Available transcriptomics data

Three data sets with baseline gene expression profiles of TB patients and the counterparts were selected for the differentially expressed analysis and machine learning (ML)-based classification studies. The data sets are: GSE107991 (21 TB, 21 latent tuberculosis infection (LTBI), and 12 Control), E-MTAB-8290 (54 TB and 127 non-TB, including presumptive symptomatic adults with negative TB diagnosis controls and with or without human immunodeficiency virus infection), and GSE101705 (28 TB and 16 LTBI)^29–32^.

### Chemicals, reagents, and consumables

The LC–MS grade ammonium formate, formic acid, methyl tertbutyl ether (MTBE), and toluene were purchased Sigma Aldrich (St. Louis, Missouri, USA). LiChroSolv® LC–MS grade solvents including water, methanol, acetonitrile, and isopropanol were purchased from Merck KGaA (Darmstadt, Germany). The SPLASH Lipidomix® Mass Spec Standard was purchased from Avanti Polar Lipids (Alabama, USA).

Acquity charged surface hybrid technology (CSH) C18 2.1 × 100 mm, 1.7 μm column and Acquity VanGuard CSH C18 2.1 × 5 mm, 1.7 μm pre-column were purchased from Waters (Milford, MA, USA).

### Sample preparation and lipid extraction

Sample preparation and lipid extraction were performed in accordance with previously established methods, with a few modifications^33,34^. In brief, 55-μL plasma samples were thawed on ice for approximately 30 min; subsequently, 5 μL were removed from each sample and pooled to obtain a quality control (QC) sample. Five microliters of the lipid internal standard mixture were injected into each sample (1:10, *v*/*v*) and the sample was briefly vortexed. After the sample had been incubated on ice for 20 min with intermittent vortexing, 300 μL of methanol (− 20 °C) and 1000 μL of MTBE (− 20 °C) were added. The mixture was vortexed vigorously for 10 s, then incubated at 4 °C for 1 h with occasional vortexing. After the addition of 250 μL of water, vigorous vortexing for 20 s, and a 10-min incubation at 4 °C, the sample was centrifuged for 2 min at 4 °C and 14,000 *rcf*. The two supernatants (lipid fraction), each comprising 500 μL, were collected. One half was used for assessments in positive ion mode, and the other half was used for assessments in negative ion mode. The lipid fraction was completely dried in a vacuum at room temperature and stored at − 80 °C until needed.

### Instrumental conditions for untargeted lipid profiling

An Acquity charged surface hybrid technology C18 column (2.1 × 100 mm, 1.7 μm) and Acquity VanGuard charged surface hybrid technology C18 pre-column (2.1 × 5 mm, 1.7 μm) were used for lipid separation with a binary gradient elution as described in Supplementary Table S2. A Shimadzu Nexera LC system (Kyoto, Japan) was utilized for the experiment. Lipid extracts were resuspended in methanol/toluene (9:1, *v*/*v*) and kept at 4 °C in an autosampler. The injected volume was ion-mode- and data-acquisition-dependent. From 200 μL of resuspended volume, 1 μL (scan profiling) and 2 μL (information-dependent acquisition and SWATH-based data-independent acquisition) were injected in positive ion mode; 3 μL (scan profiling) and 6 μL (information-dependent acquisition and SWATH) were injected in negative ion mode. The separated lipid ions were analyzed using an X500R QTOF with a Turbo V™ ion source with a TwinSpray probe (SCIEX, MA, USA). For the tandem MS analyses, either 45 eV (spread of 15 eV) or 25 eV (spread of 15 eV) were used. The MS parameters are shown in Supplementary Table S3. Mass calibration was automatically performed after every fifth injection through the instrument’s CDS system, using X500R positive or negative calibration solutions.

### Lipid data processing, alignment, and lipid annotation

Raw data (wiff files) were directly input to MS-DIAL (version 4.8) for data processing, alignment, and lipid identification. The parameters were ion mode-dependent, as described in Supplementary Table S4. The aligned data were exported for subsequent use. Post-alignment data processing was performed using MetaboAnalyst 5.0 and features with missing rates ≥ 50% were removed; otherwise, the k-nearest neighbors algorithm was used to impute the missing features^35^. Features with relative standard deviation of ≥ 25% in the pooled QC were also removed. The MS-DIAL inbuilt library and Fiehn’s lab lipidomics library were used for lipid identification^36,37^.

### Transcriptomics data processing and differential analysis

Raw counts of transcripts mapped into genes were summarized using the sum level. The annotated gene-level raw counts were normalized using Trimmed Mean of M-values. The pipeline was implemented using NetworkAnalyst 3.0^38^. Differentially expressed analysis was applied for lipid-related genes in the three transcriptome profiles (i.e., E-MTAB-8290, GSE107991, GSE101705) using two-sided unpaired t-test (rstatix package version 0.7.0, implemented in R 4.1.2). Genes with a false discovery rate (FDR) less than 0.05 were considered as significant.

### Data exploration and visualization

An unsupervised method, principal component analysis (PCA), was employed to explore and visualize the lipidome data. Prior to the analysis, the data were normalized (using the median method), log-transformed, and Pareto scaled. PCs that explained the most sample variance were plotted in a two-dimensional space (MetaboAnalyst 5.0) or three-dimensional space (R package, Plotly version 4.10.0). Heatmap and volcano plots (MetaboAnalyst 5.0) were also used for data visualization.

### Statistical analysis and modeling of lipidomic data

Prior to univariate analysis using an unpaired t-test, the data were normalized using the median method and log-transformed. An FDR of 0.05 was set as the threshold for significant features. Fold-change (FC) thresholds of 1.2, 1.5, and 2 were also tested for biomarker candidate selection. Class discrimination between the lipid profiles of the two groups was achieved using partial least squares-discriminant analysis (PLS-DA). Because the discriminant model has tuning parameters (e.g., the number of components), the optimal model was selected in a tenfold cross-validation process. The variable importance in projection (VIP) score of the PC1 of the optimal model was set at ≥ 1.2 as the threshold of important features used to detect potential biomarker candidates. Statistical analyses were conducted using MetaboAnalyst 5.0 unless stated otherwise.

### Internal and external biomarker validation

Univariate receiver operating characteristic (ROC) curve analysis was conducted to examine the potential biomarker applications of individual lipids. Random forest and linear support vector machine (SVM) were carried out to investigate the discriminatory capacity of the lipid biomarker candidates. Random forest is an ensemble method that generates many decision trees, then aggregates their outcomes to obtain greater prediction accuracy^39^. This powerful tool uses bagging and random feature selection to build multiple base learners. In SVM, a hyperplane is identified that maximizes the margin from data points. A larger margin leads to greater separation by the hyperplane, thus reducing generalization error. The performances of random forest and SVM are stable, regardless of the domain and data types.

For internal validation, the ROC curve-based exploratory analysis was utilized because it can automate important feature identification and performance evaluation. In the external validation, the biomarkers that were overlapped with the quantified lipids in the data of Cho et al. (at the fatty acyl/alkyl sum composition) were used to validate their performance in classifying TB patients from latent infection and controls^21^. All matched biomarkers were used to establish the biomarker models. The analyses were carried out in three different scenarios: TB vs. LTBI + control, TB vs. LTBI, and TB vs. control. The biomarker models using lipid-related genes in the datasets E-MTAB-8290 (54 TB, 127 control/non-TB), GSE107991 (21 TB, 12 controls, 21 LTBI), and GSE101705 (28 TB, 16 LTBI) were trained and validated using the same approach. In particular, the gene expression of matched lipid-related genes in four different data sets were utilized for the ROC-curve-based exploratory analysis. In all analyses, the area under the curve (AUC) and 95% confident interval (CI) of the best models are reported. The analyses were performed in the “Biomarker Analysis” module of MetaboAnalyst 5.0.

### Correlation network analysis

Normalized expression levels of lipid biomarker candidates were visualized by correlation network analysis in the R package corrr (version 0.4.3). The network shows variables as nodes and their association as edges. The proximity of two nodes is determined by their correlation strength; their locations (or Euclidean coordinates) are found by multidimensional scaling. This method reduces the number of data dimensions to facilitate variable visualization.

### Functional analysis

Biomarker candidate data were submitted to Lipid Ontology (LION) for lipid ontology enrichment analysis via the “LION-PCA heatmap” module^40^. In addition, lipid-gene association networks were analyzed using Lipidsig and lipid-genes were extracted^41^. For visualization, the R package ggplot2 (version 3.3.5) was used.

## Results

### Lipid profiles of TB patients are distinguishable from lipid profiles of controls

**Figure 1:**
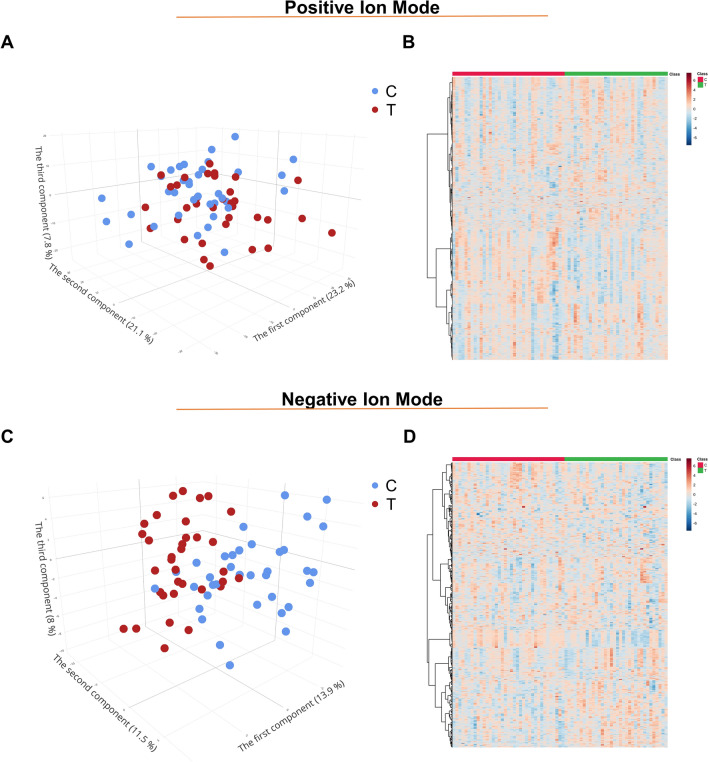
Plasma lipidome data visualization of Tuberculosis patients (N = 35) and Control (N = 37) group. (**a**) Principal components analysis 3D score plot of the two group in the positive ion mode. (**b**) Heatmap of all lipidome features between two group in the positive ion mode. (**c**) Principal components analysis 3D score plot of the two group in the negative ion mode. (**d**) Heatmap of all lipidome features between the two group in the negative ion mode. *C* control group, *T* Tuberculosis group.

PCA was performed in positive ion mode to explore sample tendencies independent of sample source. The analysis was conducted using a total of 3791 detected lipid features of TB and control samples, with and without QC samples. In the PCA scores plot with QC samples, all QC samples clustered tightly together (Fig. S1A), and the relative standard deviation of the raw total ion chromatogram among QC samples was only 6.5%. These data indicated satisfactory repeatability of the untargeted lipid profiling analysis, which allowed subsequent data analyses and interpretation. In the PCA scores plot with TB and control samples, the three first PCs explained 52.1% of the variance: 23.2%, 21.1%, and 7.8% for PCs 1, 2, and 3, respectively. The relative separation of samples into two separate groups is evident in the three-dimensional PCA plot (Fig. 1A). Heatmap analysis captured relative differences between the two groups at the feature level; differences in lipid features were relatively clear (Fig. 1B). In addition, PCA analysis, which included 762 detected features, were also conducted in negative ion mode. Similar to positive ion mode, the QC samples clustered together (relative standard deviation of total ion chromatogram: 5.6%, Fig. S1B). The three-dimensional PCA plot indicated relative separation of the samples into two groups (Fig. 1C). At the feature level in the heatmap, we could also notice a proportionately contrast between the two groups (Fig. 1D). Taken together, the data exploration analyses in positive and negative ion modes indicated considerable differences between the lipid profiles of TB patients and of controls.

### Univariate and multivariate analyses suggest numerous lipid biomarker candidates

**Figure 2:**
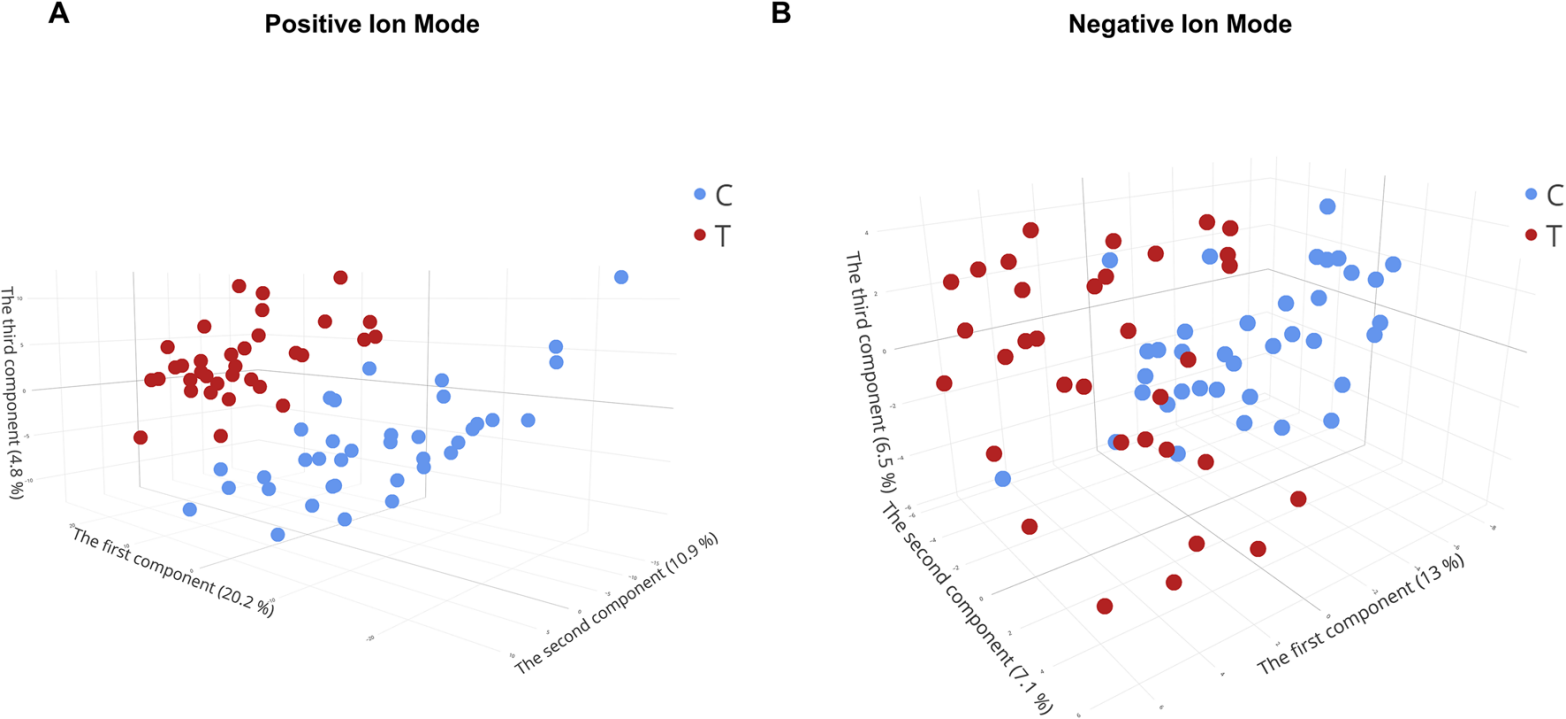
Partial least squares-discriminant analysis (PLS-DA) score plots of Tuberculosis patients and controls plasma lipidome. (**a**) PLS-DA 3D score plot of the two group in the positive ion mode. (**b**) PLS-DA 3D score plot of the two group in the negative ion mode. *C* control group, *T* Tuberculosis group.

Data exploration indicated considerable differences in lipid metabolic profiles between TB patients and controls, but a more sophisticated statistical approach was needed to identify lipids that could be regarded as biomarker candidates. Supervised investigation was conducted using PLS-DA and the lipid profiles of TB patients and controls. In positive ion mode, a PLS-DA model with five components classified the two groups with appropriate performance metrics (Fig. 2A, accuracy = 0.90, R^2^ = 0.92, and Q^2^ = 0.58). Similarly, in negative ion mode, a PLS-DA model with five components provided satisfactory classification (Fig. 2B, accuracy = 0.90, R^2^ = 0.95, and Q^2^ = 0.62) (Fig. S2A,B). The VIP score of the first PC, which explained the most sample variance, was extracted as an additional metric of biomarker candidate potential. A VIP score ≥ 1.2 was determined for 821 (21.66%) and 139 (15.71%) features in positive and negative ion modes, respectively. Finally, the random forest model demonstrated satisfactory performance in distinguishing the two groups. The cross-validated out-of-bag errors were 19.7% and 11.3% for positive and negative ion modes, respectively.

Univariate analysis using a t-test was employed to further explore potential biomarker candidates. In positive ion mode, 752 significant features (351 up- and 401 downregulated in TB patients) were found based on an FDR threshold of 0.05. Among these features, 743 (343 up- and 400 downregulated in TB patients), 404 (115 up- and 289 downregulated in TB patients), and 195 (51 up- and 144 downregulated in TB patients) exceeded the FC thresholds of 1.2, 1.5, and 2.0, respectively. In negative ion mode, 175 significant features (94 up- and 81 downregulated in TB patients) were identified with an FDR threshold of 0.05. With the FC thresholds of 1.2, 1.5, and 2.0, 156 (78 up- and 78 downregulated in TB patients), 74 (23 up- and 51 downregulated in TB patients), and 33 (8 up- and 25 downregulated in TB patients) features were selected, respectively. The volcano plots in Supplementary Fig. S3A (positive ion mode) and S3B (negative ion mode) show significant features based on the FC threshold of 1.5 and FDR threshold of 0.05.

The intersection of two criteria, a VIP score (PC1, PLS-DA model) of ≥ 1.2 and an FC (t-test) of ≥ 1.5, revealed 89 and 28 potential biomarker candidates in positive and negative ion modes, respectively. Among the selected features, 73 (positive ion mode) and 26 (negative ion mode) were successfully annotated as lipids, thus yielding 93 non-overlapping lipid biomarkers (Table 1).

**Table 1:** Statistics information of the potential biomarkers for TB versus control distinguish.

ID	Analyte	Ion mode	Regulation in TB	VIP score	Fold change	FDR	Concentration* (µM)	
							In TB	In control
1	CAR(20:4)	Positive	Up	1.488	1.642	3.16E−02	NA	NA
2	Cer(d34:1)	Positive	Up	1.640	1.531	6.74E−03	NA	NA
3	DG(40:7)	Positive	Down	1.666	0.644	8.85E−03	0.87	1.52
4	DG(40:8)	Positive	Down	2.591	0.482	9.50E−04	0.46	1.18
5	Hex2Cer(d42:2)	Positive	Up	1.623	1.554	6.99E−03	NA	NA
6	LPC(20:3)	Positive	Up	1.582	1.846	2.02E−02	0.09	0.05
7	LPC(22:4)	Positive	Up	1.821	1.727	3.30E−03	0.09	0.06
8	LPC(O-18:0)	Positive	Up	1.656	1.512	2.75E−03	0.23	0.16
9	LPC(O-18:1)^a^	Positive	Up	1.822	1.612	1.28E−03	0.53	0.35
10	LPE(O-16:1)^b^	Positive	Up	2.602	1.961	8.08E−07	0.42	0.23
11	NAE(16:1)	Positive	Down	2.413	0.524	2.92E−03	NA	NA
12	PC(34:4)^c^	Positive	Down	1.666	0.603	8.85E−03	0.47	0.88
13	PC(34:5)	Positive	Down	1.906	0.496	8.45E−03	0.56	1.11
14	PC(35:5)	Positive	Down	1.778	0.517	1.09E−02	0.50	1.01
15	PC(36:0)	Positive	Up	2.319	1.727	1.82E−06	1.96	1.15
16	PC(36:6)	Positive	Down	1.727	0.660	2.55E−03	2.51	3.80
17	PC(38:3)	Positive	Up	1.427	1.815	3.76E−02	9.14	5.30
18	PC(38:7)	Positive	Down	1.865	0.595	1.51E−03	3.96	6.20
19	PC(41:7)	Positive	Down	1.600	0.651	2.20E−02	0.27	0.42
20	PC(42:8)	Positive	Up	1.706	1.590	3.65E−03	0.80	0.47
21	PC(45:11)	Positive	Down	1.784	0.524	2.36E−02	0.52	1.00
22	PC(O-32:1)	Positive	Down	1.713	0.580	1.62E−02	0.35	0.68
23	PC(O-34:0)	Positive	Up	1.946	1.611	4.04E−04	1.50	0.97
24	PC(O-36:0)	Positive	Up	2.660	2.064	5.56E−06	0.35	0.17
25	PC(O-37:5)	Positive	Down	1.284	0.649	3.28E−02	20.68	34.06
26	PC(O-38:4)	Positive	Up	1.679	1.555	5.93E−03	5.74	3.90
27	PC(O-39:5)	Positive	Up	1.551	1.543	9.08E−03	0.25	0.15
28	PC(O-40:4)	Positive	Up	1.576	1.515	1.14E−02	0.94	0.65
29	PC(O-42:5)	Positive	Up	1.400	1.524	2.81E−02	5.07	3.56
30	PC(O-44:5)	Positive	Up	1.437	1.524	2.70E−02	7.95	5.63
31	PE(34:1)^d^	Positive	Up	1.747	1.523	5.01E−03	1.77	1.17
32	PE(36:1)^e^	Positive	Up	2.492	1.876	4.19E−06	1.25	0.76
33	PE(38:4)	Positive	Up	1.913	1.531	1.12E−04	10.70	7.53
34	PE(O-40:5)	Positive	Up	2.228	1.767	1.77E−04	0.96	0.57
35	PE(O-40:5)^f^	Positive	Up	3.386	2.739	6.02E−09	0.48	0.19
36	PI(38:5)	Positive	Up	1.879	1.862	5.35E−03	NA	NA
37	TG(36:0)	Positive	Up	2.382	7.866	6.74E−03	0.26	0.03
38	TG(38:0)	Positive	Up	2.597	7.877	2.88E−03	0.33	0.03
39	TG(40:0)	Positive	Up	2.676	9.277	3.30E−03	0.57	0.08
40	TG(42:0)	Positive	Up	2.275	5.680	1.37E−02	0.81	0.17
41	TG(42:1)	Positive	Up	2.289	6.939	2.75E−02	0.53	0.10
42	TG(42:2)	Positive	Up	2.170	13.337	4.47E−02	0.39	0.04
43	TG(51:6)	Positive	Down	1.483	0.620	2.97E−02	0.04	0.09
44	TG(52:5)	Positive	Down	1.469	0.655	3.67E−02	26.51	41.26
45	TG(52:6)	Positive	Down	2.299	0.494	2.81E−03	0.90	2.48
46	TG(54:7)	Positive	Down	1.508	0.633	2.59E−02	3.74	6.31
47	TG(54:7)	Positive	Down	1.584	0.599	3.16E−02	3.85	6.56
48	TG(54:7)	Positive	Down	1.705	0.610	1.14E−02	0.09	0.17
49	TG(54:8)	Positive	Down	2.036	0.557	4.96E−03	0.86	1.59
50	TG(54:8)	Positive	Down	1.870	0.441	1.27E−02	0.24	0.58
51	TG(55:7)	Positive	Down	1.715	0.607	1.02E−02	0.29	0.48
52	TG(56:8)	Positive	Down	2.080	0.550	2.92E−03	12.25	23.76
53	TG(56:9)	Positive	Down	2.466	0.452	1.46E−03	0.79	2.09
54	TG(56:9)	Positive	Down	2.303	0.505	1.51E−03	1.04	2.00
55	TG(56:9)	Positive	Down	1.572	0.621	3.64E−02	0.55	0.89
56	TG(57:8)	Positive	Down	1.685	0.568	1.81E−02	0.21	0.42
57	TG(57:9)	Positive	Down	1.976	0.448	8.85E−03	0.05	0.15
58	TG(58:10)	Positive	Down	1.507	0.611	4.77E−02	0.85	1.22
59	TG(58:10)	Positive	Down	2.199	0.521	2.92E−03	1.38	2.73
60	TG(58:11)	Positive	Down	2.231	0.338	1.26E−02	0.19	0.57
61	TG(58:11)	Positive	Down	2.295	0.438	5.01E−03	0.31	0.57
62	TG(58:12)	Positive	Down	2.301	0.376	5.85E−03	0.02	0.06
63	TG(58:9)	Positive	Down	1.623	0.661	1.60E−02	4.27	7.02
64	TG(58:9)	Positive	Down	1.396	0.662	4.93E−02	3.43	6.02
65	TG(60:12)	Positive	Down	2.738	0.293	2.24E−03	0.40	1.37
66	TG(60:12)	Positive	Down	1.798	0.379	3.12E−02	0.32	0.84
67	TG(60:13)	Positive	Down	2.491	0.358	3.99E−03	0.03	0.11
68	TG(60:13)	Positive	Down	2.242	0.286	1.52E−02	0.12	0.42
69	TG(62:12)	Positive	Down	1.921	0.482	1.37E−02	0.08	0.18
70	TG(62:13)	Positive	Down	2.102	0.350	1.86E−02	0.19	0.60
71	TG(62:14)	Positive	Down	2.356	0.327	9.57E−03	0.12	0.41
72	TG(62:14)	Positive	Down	1.855	0.378	3.31E−02	0.00	0.01
73	TG(64:17)	Positive	Down	2.508	0.132	2.05E−02	0.01	0.06
74	FA(14:0)	Negative	Down	1.564	0.666	5.14E−03	NA	NA
75	FA(16:1)	Negative	Down	2.408	0.531	2.27E−03	NA	NA
76	FA(18:1)	Negative	Down	2.542	0.524	3.88E−04	NA	NA
77	FA(18:2)	Negative	Down	2.878	0.426	8.41E−05	NA	NA
78	FA(18:3)	Negative	Down	2.616	0.384	1.31E−04	NA	NA
79	FA(20:1)	Negative	Down	2.353	0.515	1.31E−04	NA	NA
80	FA(20:3)	Negative	Down	2.890	0.376	2.30E−07	NA	NA
81	FA(20:4)	Negative	Down	1.988	0.557	1.53E−04	NA	NA
82	FA(20:5)	Negative	Down	2.938	0.302	6.57E−07	NA	NA
83	FA(22:4)	Negative	Down	1.974	0.542	9.61E−04	NA	NA
84	FA(22:5)	Negative	Down	3.259	0.264	1.27E−06	NA	NA
85	FA(22:6)	Negative	Down	2.699	0.376	1.09E−05	NA	NA
86	LPC(O-18:1)^a^	Negative	Up	1.710	1.567	7.46E−05	0.39	0.23
87	LPE(18:1)	Negative	Up	1.477	1.647	2.96E−03	1.10	0.66
88	LPE(O-16:1)^b^	Negative	Up	2.425	1.949	6.76E−08	0.44	0.21
89	LPE(O-18:1)	Negative	Up	2.124	1.730	3.14E−06	0.47	0.26
90	PC(34:4)^c^	Negative	Down	1.731	0.603	1.28E−03	0.10	0.15
91	PC(34:5)	Negative	Down	1.791	0.501	7.23E−03	0.03	0.06
92	PC(36:6)	Negative	Down	1.973	0.477	9.61E−04	0.19	0.35
93	PC(36:6)	Negative	Down	1.695	0.646	5.88E−04	0.58	0.79
94	PC(38:7)	Negative	Down	1.864	0.596	8.41E−05	0.90	1.28
95	PE(34:1)^d^	Negative	Up	1.740	1.612	7.85E−04	1.08	0.63
96	PE(36:1)^e^	Negative	Up	1.985	1.746	2.94E−04	1.95	1.07
97	PE(36:3)	Negative	Up	1.563	1.596	7.09E−03	1.55	0.87
98	PE(O-38:5)	Negative	Up	2.655	2.114	8.57E−10	0.91	0.32
99	PE(O-40:5)^f^	Negative	Up	3.245	2.750	2.65E−10	0.71	0.24

*TB* tuberculosis, *VIP* variable importance in projection, *FDR* false discovery rate, *NA* no information, *CAR* acylcarnitine, *Cer* ceramide, *Hex2Cer* hexosylceramide, *LPC* lysophosphatidylcholines, *LPC (O-)* Ether-linked lysophosphatidylcholines, *PC* phosphatidylcholine, *PC (O-)* Ether-linked phosphatidylcholine, *LPE* lysophosphatidylethanolamines, *LPE (O-)* Ether-linked lysophosphatidylethanolamines, *PE* phosphatidylethanolamine, *PE (O-)* Ether-linked phosphatidylethanolamine, *PI* phosphatidylinositol, *NAE* N-acetyl ethanolamine, *DG* diacylglycerol, *TG* triacylglycerol, *FA* free fatty acid.

^a–^^f^Analyte detected in both positive and negative mode.

*Single point quantification by using the peak area ratios with matched lipid class of available internal standards.

### Internal and external validation indicate satisfactory performance of lipid biomarker candidates

**Figure 3:**
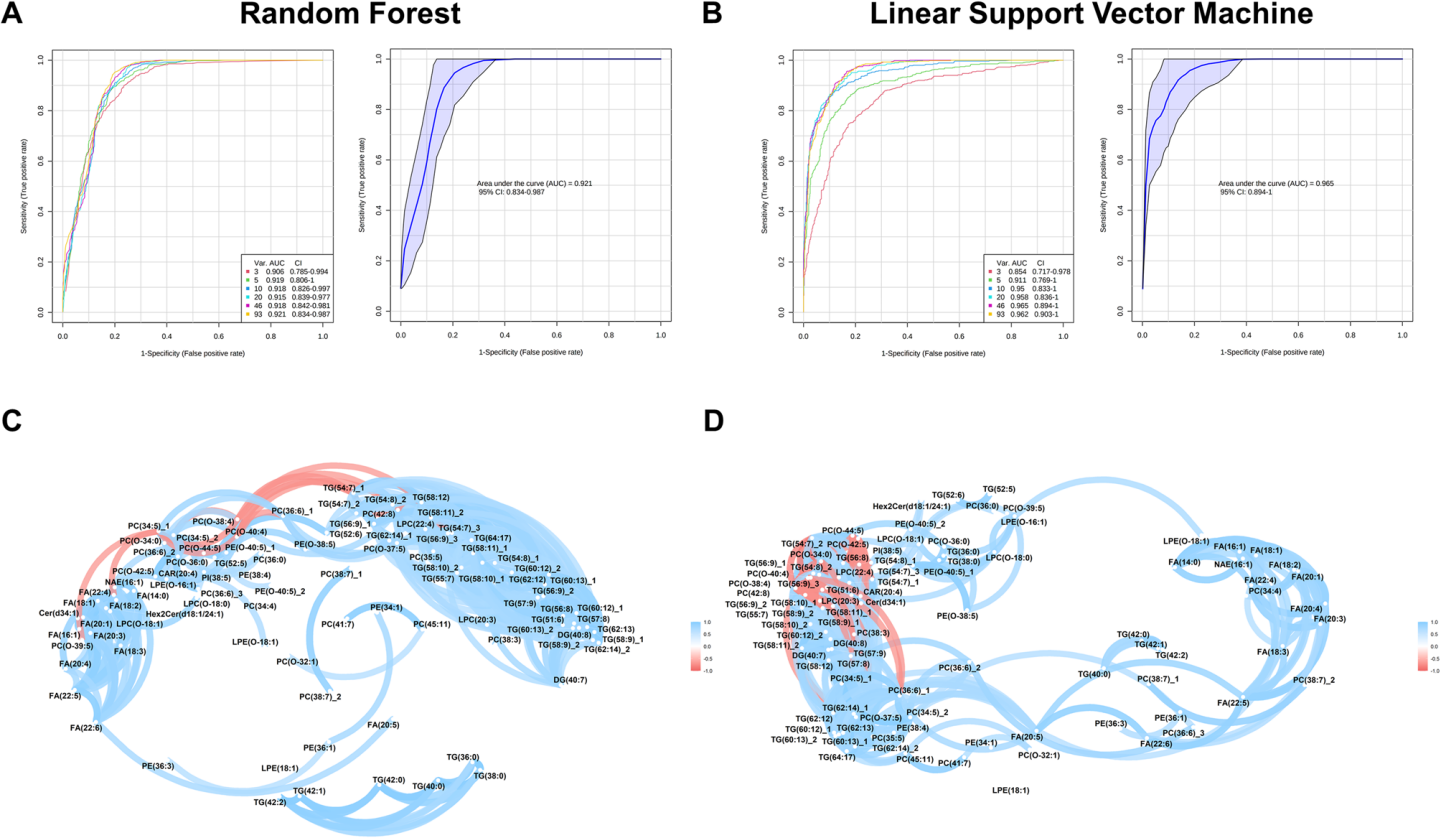
Lipid biomarkers multivariate and correlation analysis. (**a**) Random Forest predictive model of the lipid biomarkers. (**b**) Linear Support Vector Machine predictive model of the lipid biomarkers. (**c**) Correlation of the lipid biomarkers in Tuberculosis group (**d**) Correlation of the lipid biomarkers Control group. *Var* variable, *AUC* area under the curve, *CI* confidence interval, *CAR* acylcarnitine, *Cer* ceramide, *Hex2Cer* hexosylceramide, *LPC* lysophosphatidylcholines, *LPC (O-)* Ether-linked lysophosphatidylcholines, *PC* phosphatidylcholine, *PC (O-)* Ether-linked phosphatidylcholine, *LPE* lysophosphatidylethanolamines, *LPE (O-)* Ether-linked lysophosphatidylethanolamines, *PE* phosphatidylethanolamine, *PE (O-)* Ether-linked phosphatidylethanolamine, *PI* phosphatidylinositol, *NAE* N-acetyl ethanolamine, *DG* diacylglycerol, *TG* triacylglycerol, *FA* free fatty acid.

Annotated lipid biomarker candidates were first subjected to univariate biomarker analysis. The ROC curves for those candidates were significantly associated with the TB status (Supplementary Table S5). Among 93 candidate lipid biomarkers, 21 had AUC values < 0.7, whereas 72 were considered promising (AUC ≥ 0.7); of the 72, 13 were considered good (AUC ≥ 0.8) and 2 were considered excellent (AUC > 0.9). The “excellent” lipid biomarkers were two ether-linked phosphatidylethanolamines: PE(O-38:5) and PE(O-40:5). The “good” biomarker candidates were from six lipid sub-classes: two phosphatidylcholine (PC), PC(36:0) and PC(38:7); two ether-linked phosphatidylcholines (PC(O-)), PC(O-36:0) and PC(O-34:0); two ether-linked lysophosphatidylethanolamines (LPE(O-)), LPE(O-16:1) and LPE(O-18:1); two phosphatidylethanolamines (PE), (PE(36:1) and PE(38:4); one PE(O-), PE(O-40:5); and four free fatty acids (FAs), FA(20:3), FA(20:5), FA(22:5), and FA(22:6). Multivariate biomarker analysis using the random forest method revealed that models with 93 variables had the best performance (AUC = 0.921, 95% confidence interval [95% CI] 0.834–0.987) (Fig. 3A). The result of the linear SVM method was approximately similar to the result of the random forest method (Fig. 3B). Correlation analysis showed a significant linear correlation among biomarkers for both TB patients (Fig. 3C) and controls (Fig. 3D), suggesting that a small number of lipids could be used as biomarkers to differentiate TB patients from controls.

To rule out the possibility that the internal validation overestimated candidate biomarker performance, external validation was conducted using the dataset from Cho et al. (21 active TB patients, 20 patients with latent infections, 28 controls)^21^ but restricted to overlapping lipids (i.e., one LPC, 4 PCs, and 7 PC(O-)s). The validation was first conducted by dividing the samples into active TB vs. non-TB (patients with LTBI and controls) groups. In the univariate ROC analysis, six lipids exhibited an AUC ≥ 0.7. While LPC(20:3) and PC(O-34:0) were unable to differentiate between groups, the AUC of PC(O-40:4) and PC(O-42:5) was 1. Satisfactory performance (AUC 1, 95% CI 1–1) was obtained using the random forest model. Similar results were achieved in the comparison of active TB vs. LTBI or TB vs. control (Table 2). The results of the external validation partially supported the results of correlation network analysis, i.e., only a few lipids could differentiate TB from LTBI or controls.

**Table 2:** External validation performance of 12 overlapping lipid biomarkers.

Analyte		Regulation in TB		Univariate ROC analysis, AUC (CI)		
Cho et al.	Ours	Cho et al.	Our analysis	TB vs. non-TB	TB vs. LTBI	TB vs. control
lysoPC a C20:3	LPC(20:3)	Up	Up	0.581(0.421–0.735)	0.671 (0.498–0.823)	0.483 (0.327–0.638)
PC aa C34:4	PC(34:4)	Down	Down	0.899 (0.806–0.961)	0.976 (0.914–1.000)	0.854 (0.736–0.940)
PC aa C36:0	PC(36:0)	Up	Up	0.964 (0.919–0.992)	0.924 (0.819–0.986)	0.997 (0.980–1.000)
PC aa C36:6	PC(36:6)	Down	Down	0.704 (0.584–0.822)	0.857 (0.733–0.962)	0.600 (0.436–0.752)
PC aa C38:3	PC(38:3)	Down	Up	0.734 (0.584–0.864)	0.729 (0.562–0.861)	0.734 (0.566–0.879)
PC ae C32:1	PC(O-32:1)	Down	Down	0.630 (0.478–0.759)	0.656 (0.481–0.811)	0.607 (0.436–0.754)
PC ae C34:0	PC(O-34:0)	Down	Up	0.497 (0.354–0.647)	0.614 (0.431–0.783)	0.581 (0.406–0.725)
PC ae C36:0	PC(O-36:0)	Down	Up	0.660 (0.490–0.807)	0.748 (0.562–0.894)	0.607 (0.429–0.781)
PC ae C38:4	PC(O-38:4)	Up	Up	0.699 (0.535–0.846)	0.764 (0.581–0.893)	0.663 (0.479–0.809)
PC ae C40:4	PC(O-40:4)	Up	Up	1.000 (1.000–1.000)	1.000 (1.000–1.000)	1.000 (1.000–1.000)
PC ae C42:5	PC(O-42:5)	Up	Up	1.000 (1.000–1.000)	1.000 (1.000–1.000)	1.000 (1.000–1.000)
PC ae C44:5	PC(O-44:5)	Up	Up	0.479 (0.327–0.651)	0.545 (0.364–0.710)	0.447 (0.295–0.631)

*TB* tuberculosis, *LTBI* latent tuberculosis infection, *AUC* area under the curve, *CI* confidence interval, *lysoPC* lysophosphatidylcholines, *aa* diacyl, *ae* acyl-alkyl, *LPC* lysophosphatidylcholines, *PC* phosphatidylcholine, *PC (O-)* Ether-linked phosphatidylcholine, *LPE* lysophosphatidylethanolamines, *PE* phosphatidylethanolamine, *PI* phosphatidylinositol, *NAE* N-acetyl ethanolamine, *DG* diacylglycerol, *TG* triacylglycerol, *FA* free fatty acid.

### Functional analysis reveals profound lipid metabolic alterations in TB patients

**Figure 4:**
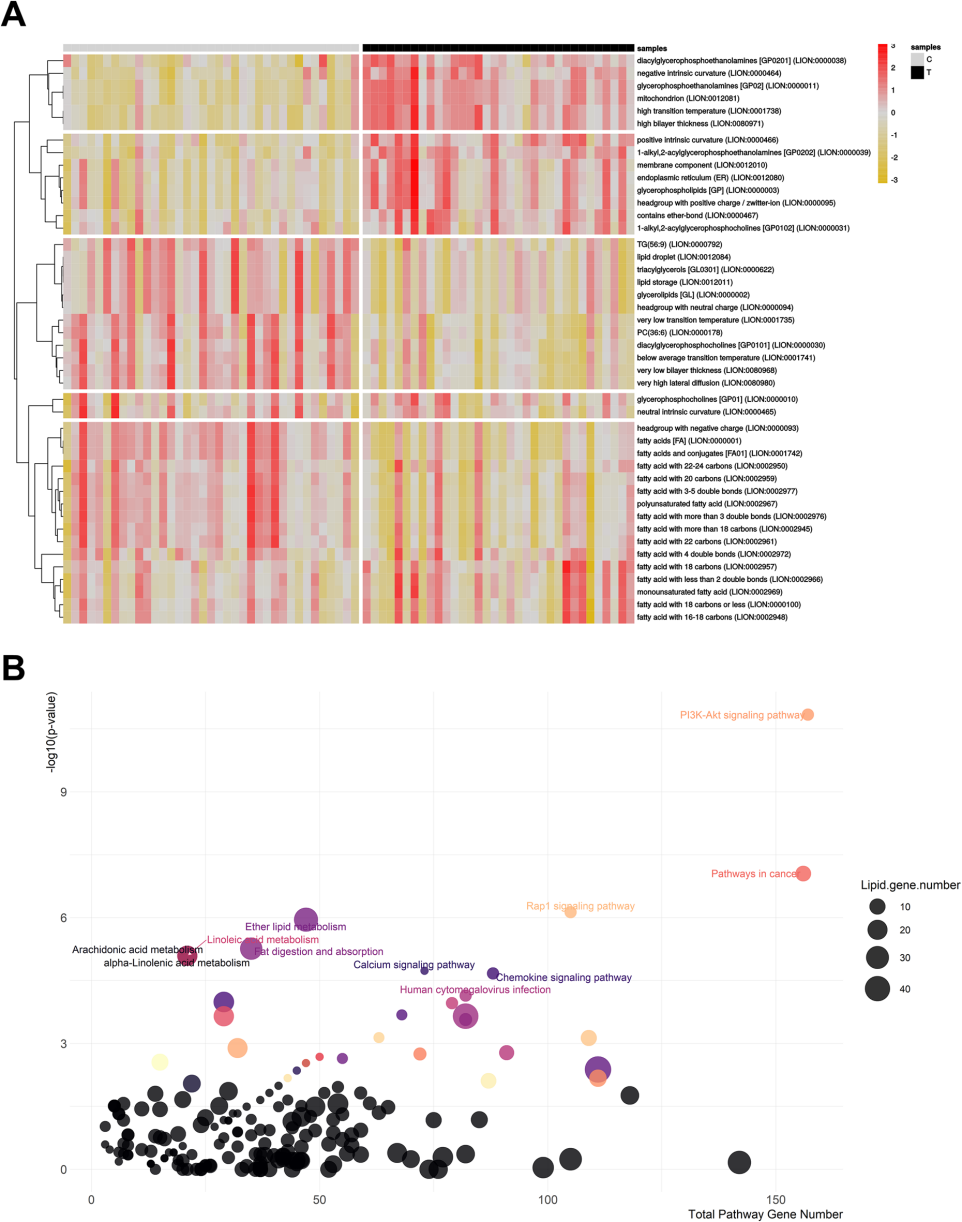
Lipid ontology enrichment and lipid-gene association network analysis. (**a**) Lipid ontology (LION) PCA-heatmap of Tuberculosis and Control group. (**b**) Bubble plot of lipid-gene association pathways. *C* control group, *T* Tuberculosis group, *PC* phosphatidylcholine, *TG* triacylglycerol, *LION* Lipid ontology.

The LION ontology results indicated that PC(O-), PC, and PE were generally enriched in the TB group, whereas FAs and triacylglycerols (TAGs) with longer acyl chains were downregulated. The TB group also exhibited enrichment of lipids associated with mitochondrion, endoplasmic reticulum, and membrane components; it showed decreased levels of LD-related lipid species (Fig. 4A).

The reported biomarker candidates belonged to 15 (sub)-classes, of which six contained ≥ 3 lipid species. Those six sub-classes were subjected to lipid-gene analysis to identify TB-associated functional dysregulation and potential gene biomarkers. As shown in Fig. 4B, numerous pathways were altered. Significantly enriched biological processes included the PI3K-Akt, Rap1, calcium, and chemokine signaling pathways. Ether lipid metabolism, fat digestion and absorption, linolenic acid metabolism, and cholesterol metabolism were also altered. The full list of altered pathways and associated genes is provided in Supplementary Table S6.

### Lipid-genes are excellent biomarkers for differentiating active TB from its counterparts

**Figure 5:**
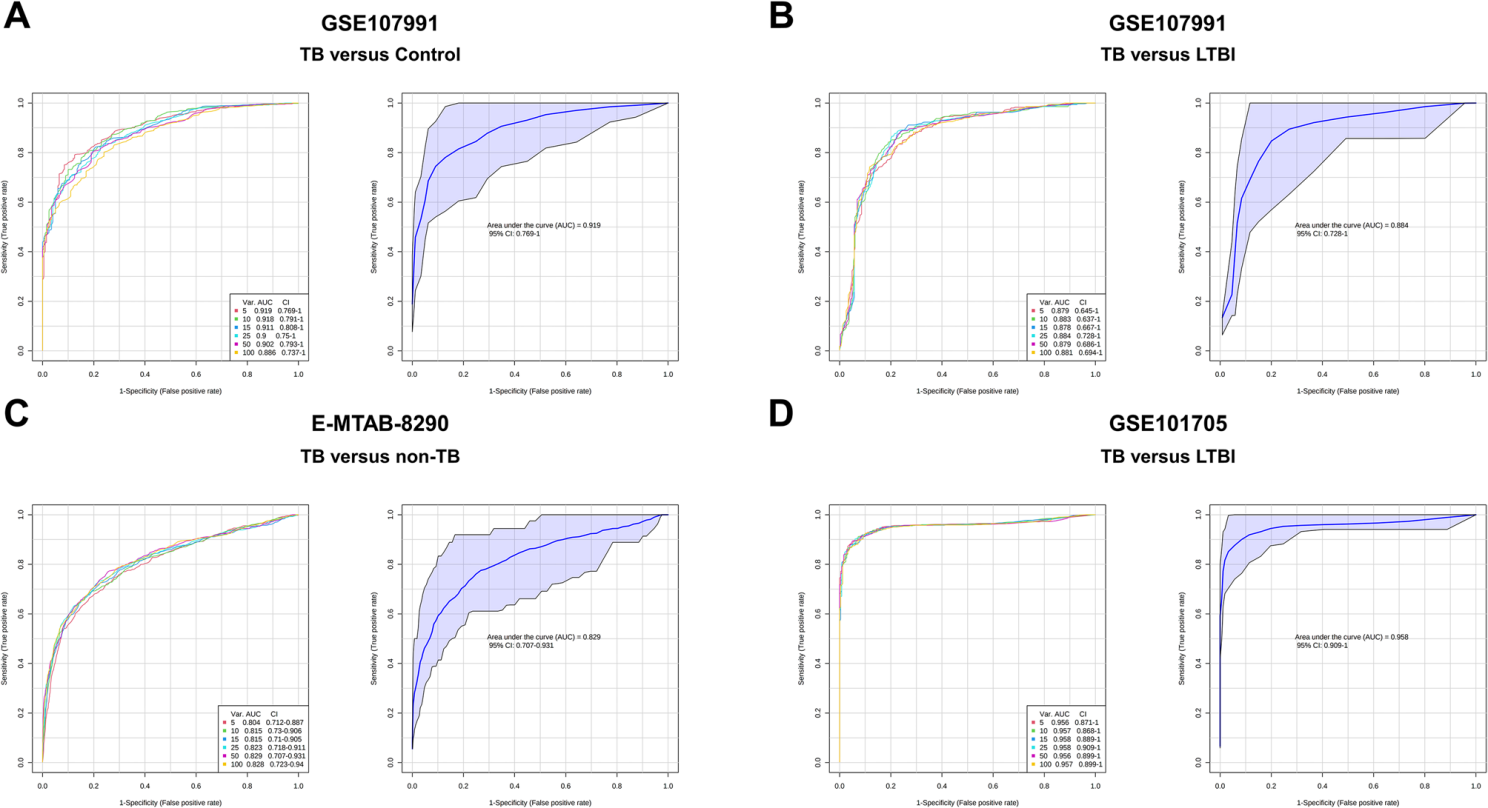
Tuberculosis (TB) and non-TB classification in three cohort by lipid-genes biomarkers using Random Forest predictive model. (**a**) Model performance (AUC = 0.919) of TB versus Control classification in GSE107991 dataset. (**b**) Model performance (AUC = 0.884) of TB versus latent tuberculosis infection (LTBI) classification in GSE107991 dataset. (**c**) Model performance (AUC = 0.829) of TB versus non-TB classification in E-MTAB-8290 dataset. (**d**) Model performance (AUC = 0.958) of TB versus Control classification in GSE101705 dataset. *Var* variable, *AUC* area under the curve, *CI* confidence interval, *TB* Tuberculosis, *LTBI* Latent tuberculosis infection.

Among dysregulated pathways detected in the lipid-gene analysis (*p* < 0.01), 162 unique genes were identified. These genes were tested for their ability to differentiate active TB from LTBI or controls in three different data sets. The random forest classifier established from the expression of lipid-related genes was able to distinguish TB from its counterparts in three different TB cohorts, with an AUC ranging from 0.829 (95% CI 0.707–0.931, E-MTAB-8290) to 0.958 (95% CI 0.909–1, GSE101705) (Fig. 5A–D). The linear SVM model showed similar results (Fig. S4A–D). However, most genes exhibited a small FC.

## Discussion

This study demonstrated differences in the lipidomes of TB patients and non-TB controls; it revealed 73 and 26 potential biomarker candidates, identified in positive and negative mode, respectively (six biomarkers were detected in both). In TB patients, the biomarkers had at least a 1.5-fold difference (in either direction). Among the significantly altered lipid sub-classes, ceramide (Cer), LPC, PC(O-), and PE were generally upregulated; certain PCs, diacylglycerols, and FAs were downregulated in TB patients. TAGs with shorter acyl chains were strongly increased in TB patients, while TAGs with longer acyl chains were decreased. The biomarkers mostly belonged to the lipid classes PC, PC(O-), PE, PE(O-), FA, and TAG, suggesting that these lipid classes are important in TB pathophysiology.

Among the putative lipid biomarkers, 12 were matched with previously reported lipid profiles that utilized a targeted approach^21^. PC(O-40:4), PC(O-42:5), PC(36:0), and PC(34:4) were prominent biomarker candidates identified by internal and external validation. Besides, our biomarkers showed concordance partially with the top biomarkers reported by Chen et al. and Han et al. in terms of lipid species. PC and PC (O-) were dominant in the top list, which suggests the importance of these lipids in differentiating TB from the controls^23,26^. However, different biological and technical factors can be attributed to the heterogeneity of the findings among studies, such as cohort characteristics, genetic background, sample treatment and instrumental data acquisition, and utilized statistical methods.

Lipid-related genes were associated with various TB pathologically comparable pathways; they also formed a distinctive signature that differentiated active TB from LTBI and other non-TB controls. Although gene expression was only subtly altered, it provided a consistent signature. Among the 162 lipid-related genes, 96 genes were found to be differentially expressed in TB versus non-TB in at least one comparison (Supplementary Table S7); these genes are presumably involved in crucial biological processes that underlie TB pathophysiology. For example, differentially expressed genes related to the biochemical regulations of PC, which is profoundly changed in TB^21,23,26^, included *CHPT1*, *LPCAT2*, *LPCAT4*, *PLA2G4A*, *PLA2G4C*, *PLD2*, *PLD4*, *MBOAT2*, *ADORA2A* and *ADORA2B*.

A significant increase in Cer(d34:1) was identified in TB patients. This biomarker has been previously reported to exhibit consistently higher levels in TB patients than in healthy individuals or patients with other respiratory diseases^23,24,42,43^. Shivakoti et al. showed that Cer(d34:1) is also related to TB treatment outcome; patients with the highest Cer levels had an increased risk of treatment failure^27^. The involvement of Cer in host immune responses against *Mtb*—via immune cell activation, phagocytosis, and other mechanisms—might explain its higher level in TB patients than in controls^24,44,45^.

Although *Mtb* relies on different carbon sources at different stages of pathogenesis, host lipids are generally the primary carbon source for *Mtb* in vivo^46^. The genome of *Mtb* laboratory strain H37Rv has > 250 genes related to lipid metabolism^47^. The infection of macrophages by *Mtb* triggers the formation of foamy macrophages through the accumulation of lipid bodies of TAGs and cholesterol esters^48,49^. Consistent with previous findings^23,26^, our study showed a decrease in TAGs in host plasma; this may be related to the uptake of host TAGs into foamy macrophages to form LDs, which can serve as nutrient sources^14,48^. While LD formation may be a host-driven immune response rather than an *Mtb*-mediated process^19^, the resulting physiological changes in *Mtb* lead to TAG accumulation, LD formation, growth reduction, decreased metabolic activity, and development of phenotypic drug resistance; these processes are associated with the persistent and non-dividing stages of *Mtb*^50^. We also found the downregulation of FAs in TB patients; this hinders the formation of longer-chain FAs that form the main components of *Mtb* cell wall lipids^51^. Fas I/II-induced elongation could partially explain the decrease in plasma FAs.

Lysophosphatidylcholine acyltransferase 2 (*LPCAT2*) induces LD accumulation in cancer patients during the onset of chemoresistance^52^. Our study is presumably the first to report an association of the upregulation of *LPCAT2* with metabolic alterations in TB patients vs. non-TB controls, suggesting that *LPCAT2* can serve as a biomarker in the diagnosis of TB.

There is also emerging evidence concerning the crucial role of metabolism in host–pathogen dynamics, with the transcription factor *PPAR* (peroxisome proliferator-activated receptor) implicated in LD buildup during inflammation and infectious diseases^53–55^. Our analysis demonstrated the enrichment of several lipid-related genes associated with *PPAR* signaling pathways; these genes include *PPARA*, *CD36*, *FABP4*, and *ACSL1*^56^. Lipid mediators, cytokines, and chemokines may act in a paracrine manner to induce LD formation^14,57^.

We also identified the involvement of lipids and lipid-related genes in chemokine signaling (*CDC42*, *FGR*, *IKBKB*, *RAC1*), ether lipid metabolism, glycerophospholipid metabolism, sphingolipid metabolism, and phospholipase D signaling pathways. In a mouse model of TB^58^ and a study of T cells from TB patients^59^, *Mtb* was found to inhibit host proinflammatory cytokine production through the PI3K-Akt signaling pathway. In the ontology analysis of lipid genes, the PI3K-Akt signaling pathway exhibited the most significant functional dysregulations in TB patients. Together, these observations support a significant role of lipid metabolism and lipid-related genes in the host immune response.

Our study had several limitations. First, its focus was on the discovery and validation of lipid biomarkers; however, there is evidence to support the use of hydrophilic metabolites (e.g., glutamic acid and glutamine) as biomarkers^21^. The combined use of these metabolites and lipids would significantly improve the detection of active TB in clinical settings. Second, other infectious respiratory diseases (e.g., community-acquired pneumonia) were not included in the lipidomics analysis. Nevertheless, some markers were able to reliably distinguish TB and LTBI. Subsequent studies should examine the differential diagnostic performance of those biomarker candidates in other infectious respiratory diseases. Third, quantitative information is available for some biomarker candidates, based on isotopically labeled internal standards at ratios relative to human plasma. However, an accurate quantification strategy (e.g., AdipoAtlas^60^) is needed to facilitate clinical application. This can be readily achieved through targeted analysis of a subset of the most promising biomarkers. Fourth, through our exploratory analysis, we enable the identification of several altered lipids and lipid genes, as well as lipid-related metabolism and immune response pathway in TB patients. Experimental studies on in vitro or animal models are required to substantiate our findings. Finally, a prospective validation cohort study with actual concentrations of lipid biomarkers is required to examine the relevance of the identified biomarkers in TB manifestations.

In summary, our study identified and validated lipid-focused biomarkers. Multiple data mining methods with lipidome and lipid-related transcript signatures were used to obtain robust biomarkers and gain new mechanistic insights into TB. Lipid species that belonged to the PC(O-), PCs, TAGs, FAs, and Cer were identified as excellent candidate biomarkers. PC(O-40:4), PC(O-42:5), PC(36:0), and PC(34:4) were externally validated and had a good performance. Additionally, our study revealed systemic and multi-omics levels of biologically relevant processes involved in host responses to *Mtb* infection. Overall, comprehensive omics analyses employing a data-driven, knowledge-based approach can support metabolism-centric biomarker discovery and validation.

## Supplementary Information

Supplementary Information.

## Data Availability

The data underlying this article cannot be shared publicly for the privacy of individuals that participated in the study. Pre-processed and imputed data will be shared by the corresponding author upon reasonable requests.

## References

[1] World Health Organization (2021). Global Tuberculosis Report 2021.

[2] Walzl G (2018). Tuberculosis: Advances and challenges in development of new diagnostics and biomarkers. Lancet Infect. Dis..

[3] Stop TB Partnership. *TB REACH Xpert Budget Estimation Tool*. Accessed 23 March 2022.

[4] World Health Organization (2014). High Priority Target Product Profiles for New Tuberculosis Diagnostics: Report of a Consensus Meeting.

[5] De Groote MA (2017). Discovery and validation of a six-marker serum protein signature for the diagnosis of active pulmonary tuberculosis. J. Clin. Microbiol..

[6] Kumar NP (2019). Plasma chemokines are biomarkers of disease severity, higher bacterial burden and delayed sputum culture conversion in pulmonary tuberculosis. Sci. Rep..

[7] Ota MO (2014). Rapid diagnosis of tuberculosis using ex vivo host biomarkers in sputum. Eur. Respir. J..

[8] Penn-Nicholson A (2020). RISK6, a 6-gene transcriptomic signature of TB disease risk, diagnosis and treatment response. Sci. Rep..

[9] Roe J (2020). Blood transcriptomic stratification of short-term risk in contacts of tuberculosis. Clin. Infect. Dis..

[10] Sambarey A (2017). Unbiased identification of blood-based biomarkers for pulmonary tuberculosis by modeling and mining molecular interaction networks. EBioMedicine.

[11] Weiner J (2018). Metabolite changes in blood predict the onset of tuberculosis. Nat. Commun..

[12] Kontsevaya I (2021). Perspectives for systems biology in the management of tuberculosis. Eur. Respir. Rev..

[13] Sweeney TE, Braviak L, Tato CM, Khatri P (2016). Genome-wide expression for diagnosis of pulmonary tuberculosis: A multicohort analysis. Lancet Respir. Med..

[14] Peyron P (2008). Foamy macrophages from tuberculous patients' granulomas constitute a nutrient-rich reservoir for *M. tuberculosis* persistence. PLoS Pathog..

[15] Park JH, Shim D, Kim KES, Lee W, Shin SJ (2021). Understanding metabolic regulation between host and pathogens: New opportunities for the development of improved therapeutic strategies against *Mycobacterium tuberculosis* infection. Front. Cell. Infect. Microbiol..

[16] Shim D, Kim H, Shin SJ (2020). *Mycobacterium tuberculosis* infection-driven foamy macrophages and their implications in tuberculosis control as targets for host-directed therapy. Front. Immunol..

[17] Saka HA, Valdivia R (2012). Emerging roles for lipid droplets in immunity and host–pathogen interactions. Annu. Rev. Cell Dev. Biol..

[18] Chai Q, Wang L, Liu CH, Ge B (2020). New insights into the evasion of host innate immunity by *Mycobacterium tuberculosis*. Cell. Mol. Immunol..

[19] Knight M, Braverman J, Asfaha K, Gronert K, Stanley S (2018). Lipid droplet formation in *Mycobacterium tuberculosis* infected macrophages requires IFN-gamma/HIF-1alpha signaling and supports host defense. PLoS Pathog..

[20] Santucci P (2019). Nitrogen deprivation induces triacylglycerol accumulation, drug tolerance and hypervirulence in mycobacteria. Sci. Rep..

[21] Cho Y (2020). Identification of serum biomarkers for active pulmonary tuberculosis using a targeted metabolomics approach. Sci. Rep..

[22] Collins JM (2018). High-resolution plasma metabolomics analysis to detect *Mycobacterium tuberculosis*-associated metabolites that distinguish active pulmonary tuberculosis in humans. PLoS ONE.

[23] Han YS (2021). Identification of potential lipid biomarkers for active pulmonary tuberculosis using ultra-high-performance liquid chromatography-tandem mass spectrometry. Exp. Biol. Med..

[24] Lau SK (2015). Metabolomic profiling of plasma from patients with tuberculosis by use of untargeted mass spectrometry reveals novel biomarkers for diagnosis. J. Clin. Microbiol..

[25] Vrieling F (2018). Patients with concurrent tuberculosis and diabetes have a pro-atherogenic plasma lipid profile. EBioMedicine.

[26] Chen JX (2021). Novel therapeutic evaluation biomarkers of lipid metabolism targets in uncomplicated pulmonary tuberculosis patients. Signal Transduct. Target. Ther..

[27] Shivakoti R (2022). Host lipidome and tuberculosis treatment failure. Eur. Respir. J..

[28] Phuoc Long N (2022). Molecular perturbations in pulmonary tuberculosis patients identified by pathway-level analysis of plasma metabolic features. PLoS ONE.

[29] Johnson WE (2021). Comparing tuberculosis gene signatures in malnourished individuals using the TBSignatureProfiler. BMC Infect. Dis..

[30] Leong S (2018). Existing blood transcriptional classifiers accurately discriminate active tuberculosis from latent infection in individuals from south India. Tuberculosis.

[31] Singhania A (2018). A modular transcriptional signature identifies phenotypic heterogeneity of human tuberculosis infection. Nat. Commun..

[32] Turner CT (2020). Blood transcriptional biomarkers for active pulmonary tuberculosis in a high-burden setting: A prospective, observational, diagnostic accuracy study. Lancet Respir. Med..

[33] Barupal DK (2018). Generation and quality control of lipidomics data for the Alzheimer's disease neuroimaging initiative cohort. Sci. Data.

[34] Lange M, Fedorova M (2020). Evaluation of lipid quantification accuracy using HILIC and RPLC MS on the example of NIST(R) SRM(R) 1950 metabolites in human plasma. Anal. Bioanal. Chem..

[35] Pang Z (2021). MetaboAnalyst 5.0: Narrowing the gap between raw spectra and functional insights. Nucleic Acids Res..

[36] Tsugawa H (2020). A lipidome atlas in MS-DIAL 4. Nat. Biotechnol..

[37] Kind T (2013). LipidBlast in silico tandem mass spectrometry database for lipid identification. Nat. Methods.

[38] Zhou G (2019). NetworkAnalyst 3.0: A visual analytics platform for comprehensive gene expression profiling and meta-analysis. Nucleic Acids Res..

[39] Breiman L (2001). Random forests. Mach. Learn..

[40] Molenaar MR (2019). LION/web: A web-based ontology enrichment tool for lipidomic data analysis. Gigascience.

[41] Lin WJ (2021). LipidSig: A web-based tool for lipidomic data analysis. Nucleic Acids Res..

[42] Andreas NJ (2020). Performance of metabonomic serum analysis for diagnostics in paediatric tuberculosis. Sci. Rep..

[43] Lam CW, Law CY (2014). Untargeted mass spectrometry-based metabolomic profiling of pleural effusions: Fatty acids as novel cancer biomarkers for malignant pleural effusions. J. Proteome. Res..

[44] Anes E (2003). Selected lipids activate phagosome actin assembly and maturation resulting in killing of pathogenic mycobacteria. Nat. Cell Biol..

[45] Chackerian A, Alt J, Perera V, Behar SM (2002). Activation of NKT cells protects mice from tuberculosis. Infect. Immun..

[46] Gouzy A (2013). *Mycobacterium tuberculosis* nitrogen assimilation and host colonization require aspartate. Nat. Chem. Biol..

[47] Cole ST (1998). Deciphering the biology of *Mycobacterium tuberculosis* from the complete genome sequence. Nature.

[48] Daniel J, Maamar H, Deb C, Sirakova TD, Kolattukudy PE (2011). *Mycobacterium tuberculosis* uses host triacylglycerol to accumulate lipid droplets and acquires a dormancy-like phenotype in lipid-loaded macrophages. PLoS Pathog..

[49] Pandey AK, Sassetti CM (2008). Mycobacterial persistence requires the utilization of host cholesterol. Proc. Natl. Acad. Sci. USA.

[50] Gengenbacher M, Kaufmann SH (2012). *Mycobacterium tuberculosis*: Success through dormancy. FEMS Microbiol. Rev..

[51] Smith JL, Sherman DH (2008). An enzyme assembly line. Science.

[52] Cotte AK (2018). Lysophosphatidylcholine acyltransferase 2-mediated lipid droplet production supports colorectal cancer chemoresistance. Nat. Commun..

[53] Almeida PE, Carneiro AB, Silva AR, Bozza PT (2012). PPARgamma expression and function in mycobacterial infection: Roles in lipid metabolism, immunity, and bacterial killing. PPAR Res..

[54] Almeida PE (2009). *Mycobacterium bovis bacillus* Calmette-Guerin infection induces TLR2-dependent peroxisome proliferator-activated receptor gamma expression and activation: Functions in inflammation, lipid metabolism, and pathogenesis. J. Immunol..

[55] Mahajan S (2012). *Mycobacterium tuberculosis* modulates macrophage lipid-sensing nuclear receptors PPARgamma and TR4 for survival. J. Immunol..

[56] Szatmari I (2007). PPARgamma regulates the function of human dendritic cells primarily by altering lipid metabolism. Blood.

[57] Dawa S (2021). Inhibition of granuloma triglyceride synthesis imparts control of *Mycobacterium tuberculosis* through curtailed inflammatory responses. Front. Immunol..

[58] Liu Y, Li JY, Chen ST, Huang HR, Cai H (2016). The rLrp of *Mycobacterium tuberculosis* inhibits proinflammatory cytokine production and downregulates APC function in mouse macrophages via a TLR2-mediated PI3K/Akt pathway activation-dependent mechanism. Cell. Mol. Immunol..

[59] Zhang X (2017). Inhibition of the PI3K-Akt-mTOR signaling pathway in T lymphocytes in patients with active tuberculosis. Int. J. Infect. Dis..

[60] Lange M (2021). AdipoAtlas: A reference lipidome for human white adipose tissue. Cell Rep. Med..

