# Supplementary material for: Comprehensive lipid and lipid-related gene investigations of host immune responses to characterize metabolism-centric biomarkers for pulmonary tuberculosis
Source: Sci Rep. 2022 Aug 4;12:13395. doi: 10.1038/s41598-022-17521-4 (PMC9352691; doi:10.1038/s41598-022-17521-4)
Supplement: Supplementary file 1 — Supplementary Information. [file 41598_2022_17521_MOESM1_ESM.pdf]

Comprehensive Lipid and Lipid-Related Gene Investigations of Host Immune Responses to Characterize Metabolism-Centric Biomarkers for Pulmonary Tuberculosis

Nguyen Phuoc Long[1,2,*], Nguyen Ky Anh[1,2,*], Nguyen Thi Hai Yen[1,2], Nguyen Ky Phat[1,2], Seongoh Park[3], Vo Thuy Anh Thu[1,2], Yong-Soon Cho[1,2], Jae-Gook Shin[1,2,4], Jee Youn Oh[5,#], Dong Hyun Kim[1,#]

[1]Department of Pharmacology and PharmacoGenomics Research Center, Inje University College of Medicine, Busan, Republic of Korea

[2]Center for Personalized Precision Medicine of Tuberculosis, Inje University College of Medicine, Busan, Republic of Korea

[3]Department of Statistics, Sungshin Women's University, Seoul, Republic of Korea

[4]Department of Clinical Pharmacology, Inje University Busan Paik Hospital, Busan, Republic of Korea

[5]Division of Pulmonary, Allergy and Critical Care Medicine, Department of Internal Medicine, Korea University Guro Hospital, Seoul, Republic of Korea

[#]: Corresponding to: Jee Youn Oh (happymaria0101@hanmail.net) and Dong Hyun Kim (dhkim@inje.ac.kr)

[*]: These authors contributed equally to this work.

SUPPLEMENTARY FIGURES

Supplementary Fig. 1. Principle component analysis (PCA) of all lipidome features with quality control (QC) samples of active-Tuberculosis (TB) patients' group (N = 35) and control group (N = 37). (**a**) PCA score plot of all lipidome features in positive ion mode. (**b**) PCA score plot of all lipidome features in negative ion mode.

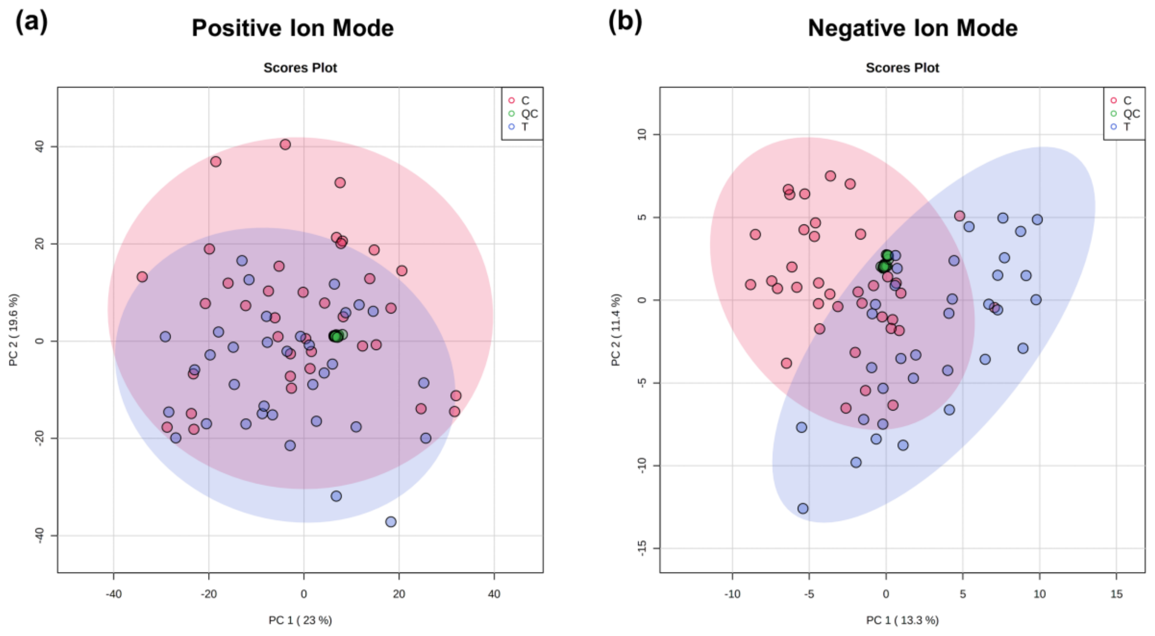

Supplementary Fig. 2. Partial Least Squares – Discriminant Analysis (PLS-DA) classification of all lipidome features cross validation. (**a**) PLS-DA cross validation in positive ion mode. (**b**) PLS-DA cross validation in negative ion mode.

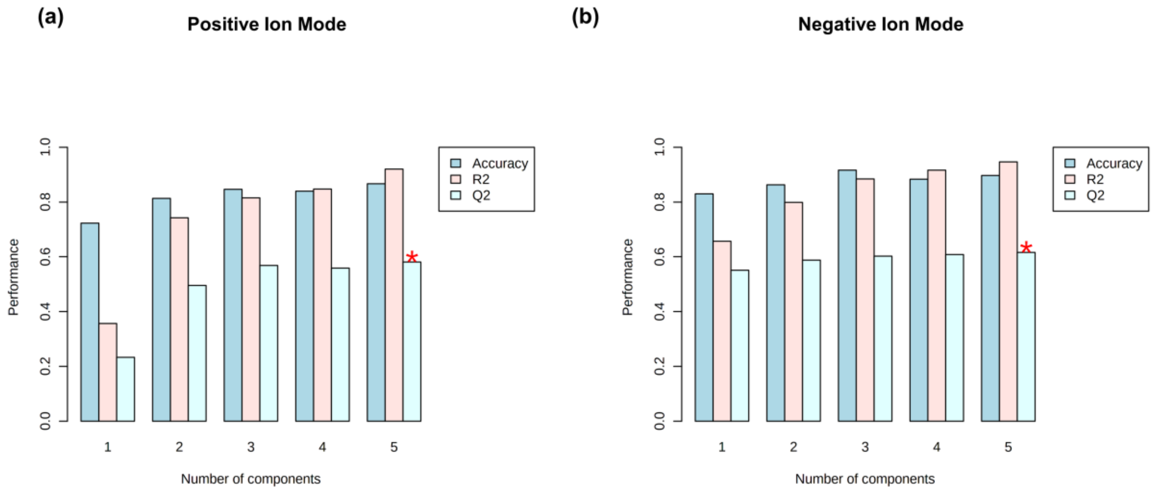

Supplementary Fig. 3. Volcano plot of significant lipidome features with fold-change of 1.5 or higher and FDR < 0.05. (**a**) Volcano plot of significant qualified lipidome features in positive ion mode. (**b**) Volcano plot of significant qualified lipidome features in negative ion mode.

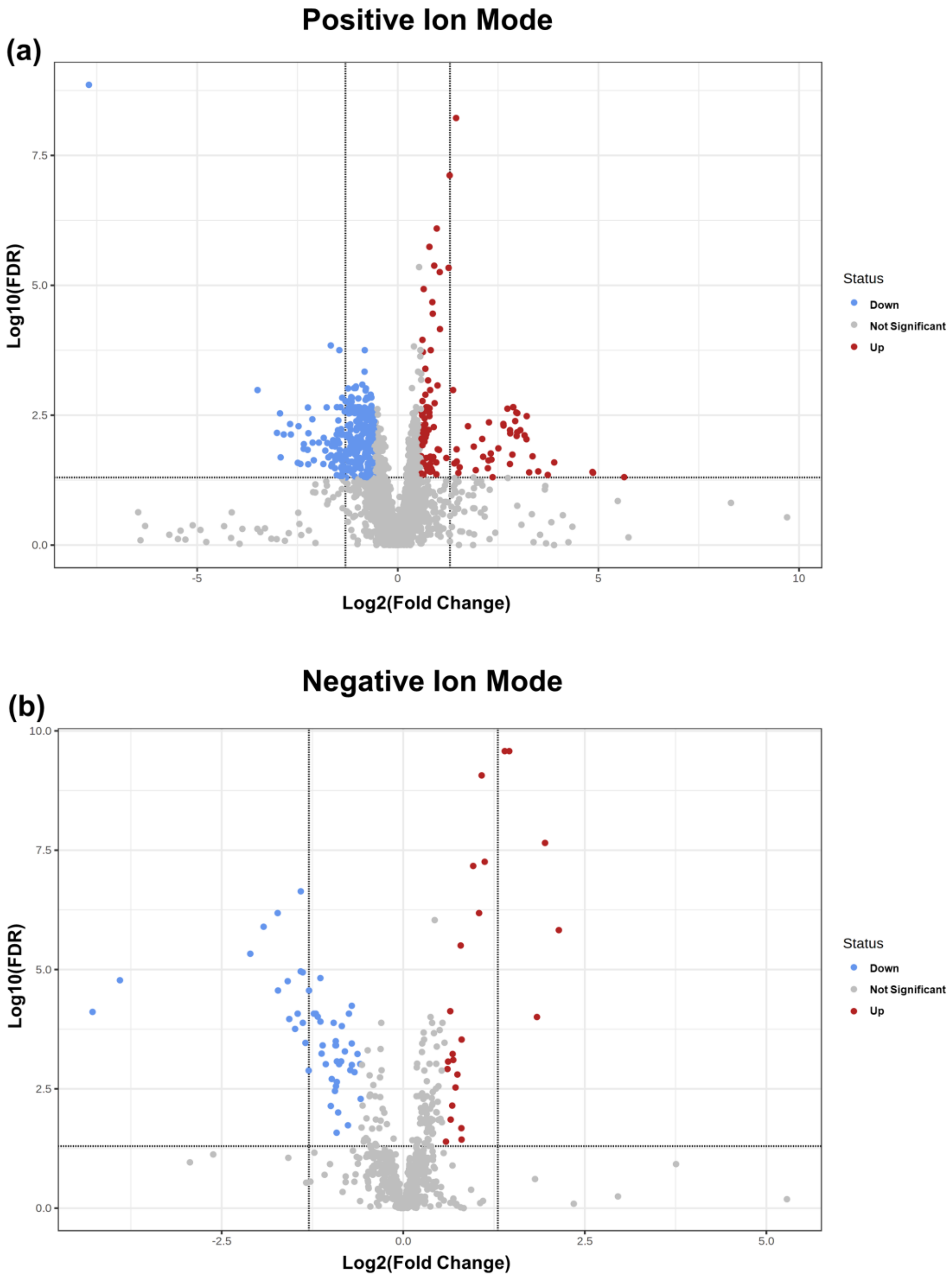

Supplementary Fig. S4. Tuberculosis (TB) and non-TB classification in three cohort by lipid-genes biomarkers using linear support vector machine (SVM) predictive model. (**a**) Model performance (AUC = 0.827) of TB versus Control classification in GSE107991 dataset. (**b**) Model performance (AUC = 0.809) of TB versus latent tuberculosis infection (LTBI) classification in GSE107991 dataset. (**c**) Model performance (AUC = 0.801) of TB versus non-TB classification in E-MTAB-8290 dataset. (**d**) Model performance (AUC = 0.956) of TB versus Control classification in GSE101705 dataset. Abbreviations: Var, variable; AUC, area under the curve; CI, confidence interval; TB, Tuberculosis; LTBI, Latent tuberculosis infection.

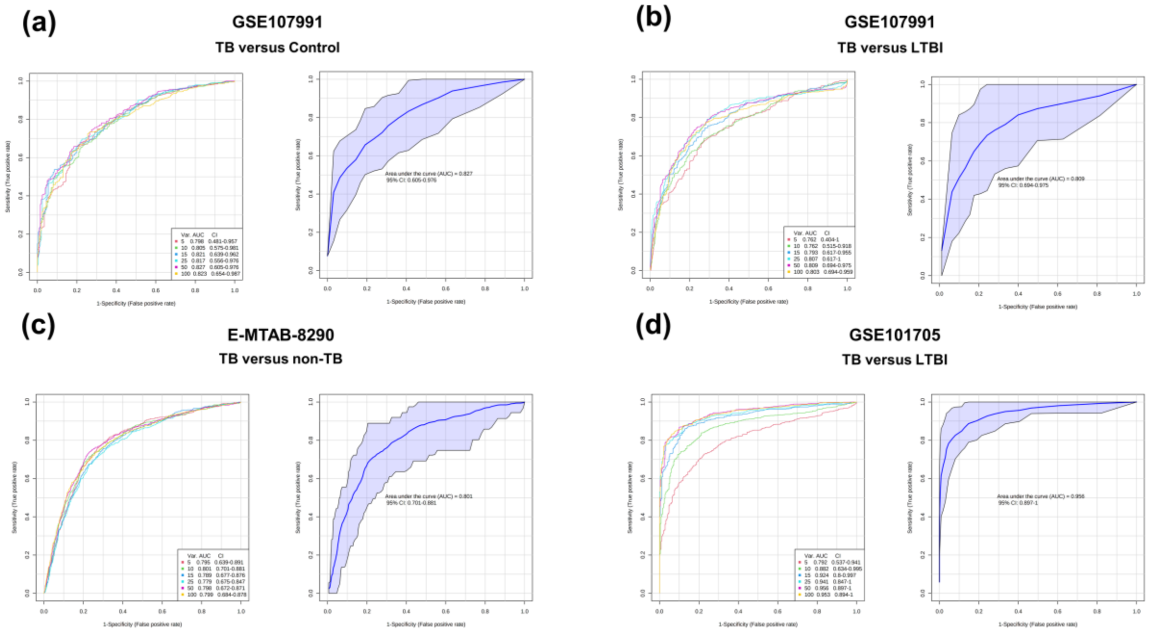

SUPPLEMENTARY TABLES

Supplementary Table S1. Clinical characteristics of the subjects.

	Active TB group	Control group	*p-value*
**Age, years, mean (range)**	60.6 ± 11 (39-86)	59.6 ± 9.6 (39-78)	0.8216[a]
**Gender, F/M (total number)**	12/23 (35)	19/18 (37)	0.1613[b]
**BMI, kg/m^2, mean (range)**	21.9 ± 2.7 (17.2 – 27.1)	25.7 ± 4.9 (19.7 – 41.0)	0.0013[a]

[a]*p-value* was calculated using Wilcoxon rank sum test.

[b]*p-value* was calculated using Fisher's exact test.

Abbreviations: TB, tuberculosis; F, female; M, male; BMI, body mass index.

Supplementary Table S2. LC gradient condition[1].

Parameter	Value
Column temperature (^{o}C)	65
Flow rate (mL/min)	0.6
Autosampler temperature (^{o}C)	4
Mobile phase A	60:40 ACN:water + 10 mM ammonium formate + 0.1% formic acid
Mobile phase B	90:10 IPA:ACN + 10 mM ammonium formate + 0.1% formic acid
**Gradients**	
0 min	15 % B
0 – 2 min	30 % B
2 – 2.5 min	48 % B
2.5 – 11 min	82 % B
11 – 11.5 min	99 % B
11.5 – 12 min	99 % B
12 – 12.1 min	15 % B
12.1 min – 15 min	15 % B
**Post-run time**	1 min

Supplementary Table S3. Specifics MS parameters for MS1, SWATH or IDA acquisition mode[1].

**Parameter**	**Value**	
**Acquisition mode**	**Simultaneous MS1 and MS/MS (SWATH)**	
ESI polarity	Positive	Negative
Curtain gas	35	35
Ion source gas 1 (psi)	60	60
Ion source gas 2 (psi)	60	60
Temperature (^{o}C)	350	350
Ion spray voltage floating (kV)	4.5	-4.5
Declustering potential (V)	80	80
MS1 accumulation time (ms)	100	100
MS1 mass range (*m/z)*	100-1700	100-1700
MS/MS accumulation time (ms)	10	10
Q1 window (Da)	20	20
SWATH mass range (*m/z)*	300-1100	300-1100
Number of SWATH-MS/MS experiments	40	40
MS/MS mass range (*m/z)*	80-1100	80-1100
**Mass calibration**	Once every 5 injections via X500 calibration solution positive/negative calibration	

Parameter	Value	
**Acquisition mode**	**IDA**	
ESI polarity	Positive	Negative
Curtain gas	35	35
Ion source gas 1 (psi)	60	60
Ion source gas 2 (psi)	60	60
Temperature (ºC)	350	350
Ion spray voltage floating (kV)	4.5	-4.5
Declustering potential (V)	80	80
MS1 accumulation time (ms)	250	250
MS1 mass range (*m/z)*	100-1700	100-1700
MS/MS accumulation time (ms)	10	10
MS/MS mass range (*m/z)*	80-1100	80-1100
Number of dependent MS/MS scan	10	10
**Mass calibration**	Once every 5 injections via X500 calibration solution positive/negative calibration (inbuilt CDS)	

Supplementary Table S4. MS-DIAL data processing, alignment and lipid annotation.

Parameters	Value	
	**Positive ion mode**	**Negative ion mode**
**Data processing**		
MS1 tolerance (Da)	0.01	0.01
MS2 tolerance (Da)	0.05	0.05
Maximum charged number	2	2
**Peak detection**		
Minimum peak height **(amplitude)**	500	500
Mass slice width (Da)	0.05	0.05
Smoothing method	Linear weighted moving average	
Smoothing level (scan)	3	3
Minimum peak width (scan)	5	5
**MS2**		
Sigma window value	0.5	0.5
MS/MS abundance cut off (amplitude)	0	0
**Identification**		
Retention time tolerance (min)	100	100
Accurate mass tolerance (MS1) (Da)	0.03	0.03
Accurate mass tolerance (MS2) (Da)	0.05	0.05
Identification score cut off (%)	70	70
**Adduct**	[M+H]+, [M+NH4]+, [M+Na]+, [M+K]+, [M+H-H2O]+	[M-H]-, [M-H2O-H]-, [M+Na-2H]-,[M+Cl]-

Alignment

Retention time tolerance (min)		0.05
MS1 tolerance (Da)		0.025
Blank filter: sample average / blank average	5-fold change	

Peak annotation process was executed using accurate mass and MS/MS matching in either SWATH acquisition mode (45 eV, 25 eV) or IDA (45 eV, 25 eV) to maximize lipidome coverage. For annotation, an ion mass accuracy of at least 900 was considered (preferably ≥ 950) along with the reverse dot product score (preferably ≥ 700), MS/MS pattern, and dot product score.

Supplementary Table S5. Univariate analysis of annotated biomarker candidates.

ID	Analyte	Mode	AUC	*p-value*
1	PE(O-38:5)	Negative	0.929	3.37E-12
2	PE(O-40:5)	Positive	0.916	3.17E-12
3	LPE(O-16:1)	Positive	0.893	8.52E-10
4	PE(36:1)	Positive	0.877	6.64E-09
5	PC(36:0)	Positive	0.873	2.40E-09
6	FA(20:3)	Negative	0.871	2.11E-09
7	PC(O-36:0)	Positive	0.871	1.32E-08
8	FA(20:5)	Negative	0.860	7.75E-09
9	FA(22:5)	Negative	0.854	1.83E-08
10	LPE(O-18:1)	Negative	0.839	5.36E-08
11	PE(O-40:5)	Positive	0.824	9.82E-07
12	PE(38:4)	Positive	0.823	4.14E-07
13	FA(22:6)	Negative	0.822	2.15E-07
14	PC(38:7)	Negative	0.816	2.97E-06
15	PC(O-34:0)	Positive	0.807	2.56E-06
16	FA(18:2)	Negative	0.789	2.82E-06
17	TG(56:9)	Positive	0.789	1.89E-05
18	TG(60:12)	Positive	0.789	4.26E-05
19	FA(20:1)	Negative	0.788	6.36E-06
20	DG(40:8)	Positive	0.788	8.27E-06
21	FA(20:4)	Negative	0.786	7.83E-06
22	FA(18:3)	Negative	0.784	6.33E-06

23	PC(38:7)	Positive	0.777	2.07E-05
24	LPC(O-18:1)	Positive	0.775	1.45E-05
25	TG(38:0)	Positive	0.771	7.68E-05
26	TG(56:9)	Positive	0.770	2.04E-05
27	LPC(O-18:0)	Positive	0.769	6.53E-05
28	PE(34:1)	Positive	0.768	2.05E-04
29	FA(18:1)	Negative	0.766	2.69E-05
30	TG(56:8)	Positive	0.766	8.38E-05
31	PC(36:6)	Negative	0.764	8.95E-05
32	PI(38:5)	Positive	0.764	2.37E-04
33	TG(52:6)	Positive	0.762	7.21E-05
34	TG(58:12)	Positive	0.762	2.72E-04
35	PC(36:6)	Negative	0.762	4.71E-05
36	PC(36:6)	Positive	0.762	5.77E-05
37	TG(58:10)	Positive	0.754	8.15E-05
38	FA(22:4)	Negative	0.751	8.96E-05
39	TG(54:8)	Positive	0.750	1.96E-04
40	PC(42:8)	Positive	0.750	1.22E-04
41	TG(58:11)	Positive	0.750	2.05E-04
42	PC(34:4)	Positive	0.746	5.84E-04
43	PE(36:3)	Negative	0.745	1.03E-03
44	NAE(16:1)	Positive	0.743	8.43E-05
45	TG(60:13)	Positive	0.743	1.37E-04
46	LPC(22:4)	Positive	0.741	1.05E-04
47	TG(58:11)	Positive	0.741	1.11E-03

48	LPE(18:1)	Negative	0.738	3.54E-04
49	TG(36:0)	Positive	0.738	3.58E-04
50	PC(O-38:4)	Positive	0.733	2.85E-04
51	DG(40:7)	Positive	0.728	5.83E-04
52	Cer(d34:1)	Positive	0.727	3.59E-04
53	FA(16:1)	Negative	0.724	2.59E-04
54	PC(34:5)	Positive	0.722	5.44E-04
55	Hex2Cer(d18:1/24:1)	Positive	0.718	3.84E-04
56	TG(62:14)	Positive	0.718	6.82E-04
57	TG(57:9)	Positive	0.717	5.90E-04
58	TG(54:7)	Positive	0.715	9.56E-04
59	TG(55:7)	Positive	0.715	7.45E-04
60	PC(O-39:5)	Positive	0.715	6.28E-04
61	FA(14:0)	Negative	0.711	7.07E-04
62	TG(54:8)	Positive	0.711	1.12E-03
63	TG(57:8)	Positive	0.711	1.91E-03
64	PC(34:5)	Negative	0.710	1.06E-03
65	PC(O-40:4)	Positive	0.709	9.57E-04
66	TG(51:6)	Positive	0.707	4.25E-03
67	TG(62:12)	Positive	0.707	1.25E-03
68	TG(40:0)	Positive	0.705	1.05E-04
69	TG(60:13)	Positive	0.704	1.48E-03
70	TG(64:17)	Positive	0.703	2.31E-03
71	PC(O-32:1)	Positive	0.700	1.64E-03
72	TG(58:9)	Positive	0.700	1.60E-03

73	TG(52:5)	Positive	0.698	6.13E-03
74	TG(54:7)	Positive	0.698	4.72E-03
75	PC(35:5)	Positive	0.697	8.55E-04
76	TG(62:13)	Positive	0.696	1.97E-03
77	PC(41:7)	Positive	0.694	2.56E-03
78	TG(54:7)	Positive	0.692	3.38E-03
79	CAR(20:4)	Positive	0.691	4.71E-03
80	PC(45:11)	Positive	0.691	2.87E-03
81	TG(56:9)	Positive	0.687	6.03E-03
82	TG(62:14)	Positive	0.684	5.24E-03
83	TG(58:10)	Positive	0.680	9.27E-03
84	TG(58:9)	Positive	0.680	9.72E-03
85	TG(42:0)	Positive	0.679	1.25E-03
86	PC(O-42:5)	Positive	0.677	3.91E-03
87	LPC(20:3)	Positive	0.675	2.23E-03
88	PC(O-37:5)	Positive	0.674	5.09E-03
89	PC(O-44:5)	Positive	0.674	3.63E-03
90	TG(60:12)	Positive	0.674	4.60E-03
91	TG(42:1)	Positive	0.671	3.75E-03
92	PC(38:3)	Positive	0.656	6.40E-03
93	TG(42:2)	Positive	0.654	8.39E-03

Supplementary Table 6. Significant disturbed pathways and associated genes defined using KEGG based lipid-related gene enrichment analysis.

ID	Pathways	Lipid gene number	Pathway gene number	-Log10 (*p-value*)	Lipid gene symbol
1	PI3K-Akt signaling pathway	4	157	10.84	*FASLG*, *IKBKB*, *RAC1*, *TLR4*
2	Pathways in cancer	9	156	7.05	*CDC42*, *FASLG*, *IKBKB*, *MAPK10*, *MAPK8*, *MAPK9*, *PLD1*, *PLD2*, *RAC1*
3	Rap1 signaling pathway	4	105	6.13	*ADORA2A*, *ADORA2B*, *CDC42*, *RAC1*
4	Ether lipid metabolism	35	47	5.95	*CEPT1*, *CHPT1*, *JMJD7-PLA2G4B*, *LPCAT1*, *LPCAT2*, *LPCAT4*, *PAFAH1B1*, *PAFAH1B2*, *PAFAH1B3*, *PLA2G10*, *PLA2G12A*, *PLA2G12B*, *PLA2G1B*, *PLA2G2A*, *PLA2G2C*, *PLA2G2D*, *PLA2G2E*, *PLA2G2F*, *PLA2G3*, *PLA2G4A*, *PLA2G4B*, *PLA2G4C*, *PLA2G4D*, *PLA2G4E*, *PLA2G4F*, *PLA2G5*, *PLA2G6*, *PLAAT3*, *PLB1*, *PLD1*, *PLD2*, *PLD3*, *PLD4*, *SELENOI*, *TMEM86B*
5	Fat digestion and absorption	28	35	5.26	*APOA1*, *APOA4*, *APOB*, *CD36*, *CEL*, *CLPS*, *DGAT1*, *DGAT2*, *FABP1*, *FABP2*, *LIPF*, *MOGAT3*, *MTTP*, *PLA2G10*, *PLA2G12A*,

					PLA2G12B, *PLA2G1B*, *PLA2G2A*, *PLA2G2C*, *PLA2G2D*, *PLA2G2E*, *PLA2G2F*, *PLA2G3*, *PLA2G5*, *PNLIP*, *PNLIPRP*, *PNLIPRP2*, *SCARB1*
6	Alpha-Linolenic acid metabolism	21	21	5.09	*JMJD7-PLA2G4B*, *PLA2G10*, *PLA2G12A*, *PLA2G12B*, *PLA2G1B*, *PLA2G2A*, *PLA2G2C*, *PLA2G2D*, *PLA2G2E*, *PLA2G2F*, *PLA2G3*, *PLA2G4A*, *PLA2G4B*, *PLA2G4C*, *PLA2G4D*, *PLA2G4E*, *PLA2G4F*, *PLA2G5*, *PLA2G6*, *PLAAT3*, *PLB1*
7	Arachidonic acid metabolism	21	21	5.09	*JMJD7-PLA2G4B*, *PLA2G10*, *PLA2G12A*, *PLA2G12B*, *PLA2G1B*, *PLA2G2A*, *PLA2G2C*, *PLA2G2D*, *PLA2G2E*, *PLA2G2F*, *PLA2G3*, *PLA2G4A*, *PLA2G4B*, *PLA2G4C*, *PLA2G4D*, *PLA2G4E*, *PLA2G4F*, *PLA2G5*, *PLA2G6*, *PLAAT3*, *PLB1*
8	Linoleic acid metabolism	21	21	5.09	*JMJD7-PLA2G4B*, *PLA2G10*, *PLA2G12A*, *PLA2G12B*, *PLA2G1B*, *PLA2G2A*, *PLA2G2C*, *PLA2G2D*, *PLA2G2E*, *PLA2G2F*, *PLA2G3*, *PLA2G4A*, *PLA2G4B*, *PLA2G4C*, *PLA2G4D*, *PLA2G4E*, *PLA2G4F*, *PLA2G5*, *PLA2G6*, *PLAAT3*, *PLB1*
9	Calcium signaling pathway	2	73	4.73	*ADORA2A*, *ADORA2B*
10	Chemokine signaling pathway	4	88	4.67	*CDC42*, *FGR*, *IKBKB*, *RAC1*

11	Human cytomegalovirus infection	4	82	4.14	*FASLG*, *IKBKB*, *RAC1*, *TNF*
12	Cholesterol metabolism	22	29	3.99	*APOA1*, *APOA2*, *APOA4*, *APOB*, *APOC1*, *APOC2*, *APOC3*, *APOE*, *CD36*, *CETP*, *LCAT*, *LDLR*, *LDLRAP1*, *LIPA*, *LIPC*, *LIPG*, *LPA*, *LPL*, *LRP1*, *LRP2*, *PLTP*, *SCARB1*
13	Human papillomavirus infection	4	79	3.96	*CDC42*, *FASLG*, *IKBKB*, *TNF*
14	Dopaminergic synapse	3	68	3.68	*MAPK10*, *MAPK8*, *MAPK9*
15	Necroptosis	21	29	3.65	*CHMP2A*, *CHMP2B*, *CHMP3*, *CHMP4A*, *CHMP4B*, *CHMP4C*, *CHMP6*, *CHMP7*, *FASLG*, *JMJD7-PLA2G4B*, *MAPK10*, *MAPK8*, *MAPK9*, *PLA2G4A*, *PLA2G4B*, *PLA2G4C*, *PLA2G4D*, *PLA2G4E*, *PLA2G4F*, *TLR4*, *TNF*
16	Glycerophospholipid metabolism	41	82	3.65	*AGPAT3*, *CEPT1*, *CHPT1*, *JMJD7-PLA2G4B*, *LCAT*, *LPCAT1*, *LPCAT2*, *LPCAT3*, *LPCAT4*, *MBOAT1*, *MBOAT2*, *PEMT*, *PISD*, *PLA2G10*, *PLA2G12A*, *PLA2G12B*, *PLA2G1B*, *PLA2G2A*, *PLA2G2C*, *PLA2G2D*, *PLA2G2E*, *PLA2G2F*, *PLA2G3*, *PLA2G4A*, *PLA2G4B*, *PLA2G4C*, *PLA2G4D*, *PLA2G4E*, *PLA2G4F*,

					PLA2G5, *PLA2G6*, *PLAAT3*, *PLB1*, *PLD1*, *PLD2*, *PLD3*, *PLD4*, *PTDSS1*, *PTDSS2*, *SELENOI*, *TAZ*
17	Focal adhesion	5	82	3.57	*CDC42*, *MAPK10*, *MAPK8*, *MAPK9*, *RAC1*
18	Relaxin signaling pathway	3	63	3.14	*MAPK10*, *MAPK8*, *MAPK9*
19	Regulation of actin cytoskeleton	10	109	3.13	*ACTR2*, *ACTR3*, *ARPC1A*, *ARPC2*, *ARPC3*, *ARPC4*, *ARPC5*, *CDC42*, *RAC1*, *WASL*
20	PPAR signaling pathway	20	32	2.89	*ACSBG1*, *ACSBG2*, *ACSL1*, *ACSL3*, *ACSL4*, *ACSL5*, *ACSL6*, *APOA1*, *APOA2*, *APOA5*, *APOC3*, *CD36*, *FABP1*, *FABP2*, *FABP4*, *LPL*, *OLR1*, *PLTP*, *PPARA*, *UCP1*
21	Human immunodeficiency virus 1 infection	8	91	2.78	*FASLG*, *IKBKB*, *MAPK10*, *MAPK8*, *MAPK9*, *RAC1*, *TLR4*, *TNF*
22	Phosphatidylinositol signaling system	5	72	2.75	*OCRL*, *PIK3C2A*, *PIK3C3*, *SYNJ1*, *SYNJ2*
23	mTOR signaling pathway	2	50	2.68	*IKBKB*, *TNF*
24	Neutrophil extracellular trap formation	2	50	2.68	*RAC1*, *TLR4*
25	ErbB signaling pathway	3	55	2.64	*MAPK10*, *MAPK8*, *MAPK9*
26	Synaptic vesicle cycle	12	15	2.55	*AP2A1*, *AP2A2*, *AP2B1*, *AP2M1*, *AP2S1*, *CLTA*, *CLTB*, *CLTC*, *CLTCL1*, *DNM1*, *DNM2*, *DNM3*

27	Axon guidance	2	47	2.53	*CDC42*, *RAC1*
28	PD-L1 expression and PD-1 checkpoint pathway in cancer	2	47	2.53	*IKBKB*, *TLR4*
29	Endocytosis	45	111	2.38	*ACTR2*, *ACTR3*, *AMPH*, *AP2A1*, *AP2A2*, *AP2B1*, *AP2M1*, *AP2S1*, *ARPC1A*, *ARPC2*, *ARPC3*, *ARPC4*, *ARPC5*, *BIN1*, *CDC42*, *CHMP2A*, *CHMP2B*, *CHMP3*, *CHMP4A*, *CHMP4B*, *CHMP4C*, *CHMP6*, *CHMP7*, *CLTA*, *CLTB*, *CLTC*, *CLTCL1*, *DAB2*, *DNAJC6*, *DNM1*, *DNM2*, *DNM3*, *EPS15*, *HSPA8*, *LDLR*, *LDLRAP1*, *PLD1*, *PLD2*, *RAB5A*, *RAB5B*, *RAB5C*, *SH3GL1*, *SH3GL2*, *SH3GL3*, *WASL*
30	B cell receptor signaling pathway	2	45	2.35	*IKBKB*, *RAC1*
31	Phospholipase D signaling pathway	13	111	2.17	*AGPAT3*, *DNM1*, *DNM2*, *DNM3*, *JMJD7-PLA2G4B*, *PLA2G4A*, *PLA2G4B*, *PLA2G4C*, *PLA2G4D*, *PLA2G4E*, *PLA2G4F*, *PLD1*, *PLD2*
32	Sphingolipid metabolism	2	43	2.17	*SGMS1*, *SGMS2*
33	Sphingolipid signaling pathway	9	87	2.11	*MAPK10*, *MAPK8*, *MAPK9*, *PLD1*, *PLD2*, *RAC1*, *SGMS1*, *SGMS2*, *TNF*
34	Autophagy - other	14	22	2.04	*ATG12*, *ATG16L1*, *ATG4A*, *ATG4B*, *ATG4C*, *ATG4D*, *ATG5*, *BECN1*,

		GABARAP, *GABARAPL1*, *GABARAPL2*, *PIK3C3*, *PIK3R4*, *WIPI2*

Supplementary Table S7. Differentially expressed lipid-genes in 3 cohort including EMATB-8290 (TB versus non-TB), GSE107991 (TB versus Control), GSE107991 (TB versus LTBI), GSE101705 (TB versus LTBI).

Gene	Log_2FC			
	**EMATB-8290 (TB versus non-TB)**	**GSE107991 (TB versus Control)**	**GSE107991 (TB versus LTBI)**	**GSE101705 (TB versus LTBI)**
AGPAT3	0.39***	0.6***	0.41**	0.36***
CETP	0.81***	1.16*	1.07*	1.37***
LRP1	0.55***	0.77**	0.46*	0.41*
ADORA2A	-0.37***	-0.01	-0.09	-0.17
APOA2	0.89**	Not included	Not included	0.91*
BIN1	-0.38**	-0.58**	-0.53**	-0.35**
CHMP7	-0.28**	-0.44***	-0.34**	-0.35***
LPCAT4	-0.24**	-0.32*	-0.33**	-0.08
GABARAP	0.28**	0.4	0.46*	1.36***
CD36	0.56**	0.68*	0.89***	1.03***
OCRL	0.27**	0.1	0.07	-0.14
MBOAT1	0.41**	0.3	0.34*	0.19*
PLA2G4A	0.68**	0.89**	0.98***	1.01***
LIPA	0.29**	0.32	0.49*	0.26
LPCAT2	0.72**	0.91**	0.72**	0.57**
ACSL4	0.53**	0.65**	0.58**	0.57*
PLD4	-0.52**	-0.69**	-0.66**	-0.54***

SYNJ1	0.34**	0.25	0.17	-0.15
AP2A1	0.28**	0.21	0.07	0.21
CHPT1	0.68**	0.55	1.06**	0.97**
MBOAT2	0.52**	0.65**	0.58**	0.77***
PLD2	0.23**	0.24	-0.05	0.23
CHMP2B	0.29*	0.29	0.48**	0.41***
RAB5C	0.17*	0.09	0.17	0.46***
ADORA2B	0.23*	0.56	0.33	0.52**
DNM1	0.2*	0.34	0.18	0.5*
FGR	0.27*	0.35*	0.34**	0.67***
SGMS2	0.43*	0.19	0.19	0.25
LCAT	0.16*	0.14	0.07	0.59*
SH3GL3	0.56*	Not included	Not included	Not included
LDLRAP1	-0.32*	-0.56**	-0.46**	-0.34*
LDLR	0.22*	0.38*	0.16	-0.05
ARPC1A	0.09*	0.26*	0.2**	0.42***
GABARAPL2	0.26*	0.23	0.39**	0.62***
ACSL1	0.55*	0.95**	0.8**	0.59
PLA2G4C	0.26*	0.6	0.37	0.31
DNM3	0.36*	-0.15	0.16	0.21
CLTC	0.24	0.32**	0.33**	-0.16
TLR4	0.54	0.59**	0.55**	0.58*
AMPH	-0.19	2.1*	0.94	0.33
CHMP4B	-0.01	0.24*	0.28**	0.43***
ATG16L1	-0.13	-0.12	-0.26**	-0.27***

ACSL3	0.23	0.27	0.43**	0.23
ACTR3	0.15	0.23	0.31**	0.31*
ARPC3	0.15	0.18	0.38**	1.03***
BECN1	0.05	0.11	0.16**	0.08
CLTCL1	0.25	0.36	0.63**	0.74**
ARPC5	0.25	0.37	0.46**	0.45***
CHMP2A	0.12	0.22	0.37**	0.97***
RAB5A	0.12	0.24	0.3**	0.31*
CLTA	0.04	0.09	0.24**	0.68***
CDC42	0.1	0.06	0.22**	0.37***
ACTR2	0.21	0.26	0.29*	-0.14
PLA2G6	-0.17	-0.17	-0.32*	-0.16*
CHMP3	0.06	0.09	0.21*	0.77**
PTDSS2	0.01	-0.02	-0.21*	0.34
PAFAH1B2	0.08	0.11	0.19*	0
AP2S1	0.1	0.24	0.31*	0.94***
RAC1	0.03	0.1	0.22*	0.57***
AP2A2	0.03	-0.09	-0.12*	-0.11
JMJD7-PLA2G4B	0.04	-0.23	-0.32*	Not included
SCARB1	-0.09	-0.22	-0.27*	0.08
TAZ	-0.12	-0.06	-0.21*	0.36
EPS15	0.19	0.22	0.22*	-0.16
PIK3C3	0.1	0.17	0.15*	0.28**
PISD	0.3	0.37	0.32*	0.5**

CEPT1	0.01	0.13	0.19*	0.09
PPARA	0.3	-0.19	-0.18*	-0.26**
ARPC2	0.02	-0.02	0.15	0.75***
IKBKB	0.05	0.07	-0.05	-0.19***
AP2B1	0.09	0.05	0.02	-0.35***
ACSL6	-0.01	-0.42	-0.34	-0.56***
MAPK9	-0.09	-0.04	-0.05	-0.27***
PAFAH1B1	0.09	0.1	0.1	-0.3***
SYNJ2	-0.15	-0.13	0.05	-0.58***
PIK3R4	-0.03	0.05	-0.02	-0.4***
CHMP4A	-0.22	-0.06	-0.06	0.81***
MAPK8	0.02	-0.27	-0.1	-0.27***
PLA2G4B	-0.16	-0.39	-0.17	-0.75***
CLTB	-0.15	0.03	0.04	0.83***
ATG4A	0.01	-0.08	0.08	0.38**
LPL	-0.39	-1.67	-0.98	-1.85**
PLA2G2D	-0.18	-1	-0.4	-1.03**
PLD3	0.14	0.24	0.03	0.45**
DGAT2	0.21	0.26	0.29	0.74**
ARPC4	0.07	0.27	0.17	0.7**
HSPA8	-0.11	-0.19	0.003	0.35**
WASL	0.03	-0.06	-0.03	-0.27**
CHMP6	0.02	0.09	-0.02	0.61*
DGAT1	0.03	0.14	0.1	0.41*
SH3GL1	0.06	0.11	0.08	0.24*

AP2M1	0.12	0.14	0.14	0.23*
TMEM86B	0.09	0.4	0.28	0.5*
PLB1	0.2	0.06	0.34	0.6*
ATG4D	-0.09	0.13	-0.09	0.25*
RAB5B	0.1	0.02	0.09	0.13*

FC: Fold change.

*: False discovery rate (FDR) from two-sided unpaired t-test <0.05.

**: FDR from two-sided unpaired t-test <0.01.

***: FDR from two-sided unpaired t-test <0.001.

REFERENCES

1 Barupal, D. K. *et al.* Generation and quality control of lipidomics data for the alzheimer's disease neuroimaging initiative cohort. *Sci Data* **5**, 180263, doi:10.1038/sdata.2018.263 (2018).
